# Infection History Shapes Co-Epidemic Dynamics: A Transmission Source–Pathway Decomposition for COVID-19 and Influenza

**DOI:** 10.3390/microorganisms14061239

**Published:** 2026-05-30

**Authors:** Mani Dhakal, Brajendra K. Singh, Rajeev K. Azad

**Affiliations:** 1 Department of Mathematics, University of North Texas, Denton, TX 76203, USA; manidhakal@my.unt.edu; 2 The Preserve at Killian Hill, Lilburn, GA 30047, USA; brajendra98@yahoo.com; 3 Department of Biological Sciences and BioDiscovery Institute, University of North Texas, Denton, TX 76203, USA

**Keywords:** COVID-19, influenza, co-infection, transmission pathways, sequential infection, disease burden, non-pharmaceutical interventions, vaccination, cross-susceptibility

## Abstract

The concurrent circulation of SARS-CoV-2 and influenza presents a complex immunological landscape. While biological evidence suggests that prior or current infection with one virus can alter susceptibility to the other, conventional epidemiological models often obscure these effects by aggregating co-infected populations into a single compartment. This structural simplification limits our ability to quantify how infection history shapes population-level transmission dynamics. We developed a stratified, deterministic co-infection model that explicitly distinguishes between single, concurrent, and sequential infections by accounting for sequence-dependent heterogeneity in susceptibility and transmissibility. Our primary innovation is a transmission source–pathway decomposition framework that mathematically attributes the rate of new infections to its specific transmission source (i.e., which infectious subpopulation is generating the transmission: the singly-infected, co-infected, or sequentially-infected class) and transmission pathway (i.e., which susceptibility class is receiving new infections: fully susceptible individuals, or those with prior immunity, or those with active co-infection). This framework accounts for altered susceptibility and transmissibility dependent on infection history. Our model-based analysis reveals a profound, sequence-driven asymmetry in transmission. In a baseline co-epidemic scenario, COVID-19 is predominantly driven by a sequential source: individuals who contracted COVID-19 *after* recovering from influenza are estimated to account for approximately 73% of new COVID-19 cases and approximately 76% of the disease burden, as predicted by our model. Conversely, influenza transmission remains driven by singly infected individuals (approximately 96% of new influenza cases inferred using our model). This sequence-driven asymmetry was robust to changes in model structure (especially, the inclusion of an influenza latent period in a sensitivity analysis) and across scenarios of varying relative transmissibility for the two viruses. Interventions exhibit pathway-specific effects: COVID-19 vaccination, for instance, disproportionately disrupts this dominant sequential transmission engine by protecting the most immunologically vulnerable hosts. Our model-based findings suggest that infection history may be a primary driver of co-epidemic dynamics. Our framework reveals a plausible, asymmetric interaction where an initial influenza wave can fundamentally reshape the transmission landscape for COVID-19, and demonstrates how a prior COVID-19 wave may fuel subsequent influenza transmission under specific temporal conditions. These findings generate the testable hypothesis that cross-viral susceptibility is a key control point and underscore the importance of pathway-aware intervention strategies that account for infection history.

## 1. Introduction

The concurrent circulation of SARS-CoV-2 and influenza creates a complex immunological landscape characterized by potential viral interactions [1]. While biological evidence suggests that an infection with one of these respiratory viruses can significantly alter the host’s susceptibility to the other [2,3], the population-level consequences of these interactions remain poorly understood [3]. Mathematical models of influenza and SARS-CoV-2 co-circulation have successfully incorporated complex features such as vaccination, waning immunity, and age-structure [4,5,6,7,8,9]. However, they often share a critical limitation: the treatment of co-infected individuals as a single, aggregated compartment in the model. This structural simplification—a common assumption in co-infection modeling [10]—assumes all co-infected individuals are epidemiologically identical, regardless of which virus they contracted first. As these models ignore the order of infections, they cannot account for heterogeneity in susceptibility and infectiousness that depends on infection history [2,11,12].

The need to resolve these transmission dynamics is clear. While biological evidence suggests that prior infection with one virus can alter the course of a subsequent infection—ranging from viral interference to enhanced infectivity [1,13,14]—conventional models obscure these effects. Population-level studies have found mixed evidence for interactions, with some suggesting prior influenza acts as a risk factor for SARS-CoV-2 [1,13,14], while others find limited effects or short-term protection [3,15,16]. This ambiguity highlights a critical knowledge gap: the lack of a mechanistic framework capable of quantifying how the sequence or order of an infection with one virus reshapes the population-level transmission landscape for another. The existing co-infection models cannot attribute new infections to their source subpopulation, nor can they infer the immunological pathway(s) through which the infections occur—the two pressing issues that our proposed decomposition framework is designed to address.

To address this gap, we developed a stratified co-infection model that allows us to decompose the population-level rate of new infections into its constituent parts. In practice, our framework mathematically attributes the incidence rate to a specific transmission source class (e.g., the singly-infected or co-infected subpopulation) and infers the transmission pathway through which those infections occur (e.g., infection entering the fully susceptible class versus the class recovered from a prior infection). By explicitly stratifying the population by infection history, our model provides a novel approach for understanding the relative contributions of different subpopulations to the overall epidemic dynamics.

This framework addresses the limitations of the existing models by providing the resolution needed to investigate sequence-dependent phenomena. Rather than treating co-infection as a monolithic state, our approach allows us to quantify how an initial wave of one pathogen creates a new, immunologically distinct population that can either resist or facilitate the spread of the second. By disentangling the co-epidemic into these mechanistic components, we can identify which pathways are most critical for transmission, and which interventions are best suited to disrupt them, offering a more granular understanding for public health planning. Note that our model focuses on influenza A/B viruses (collectively referred to as “influenza” throughout), as these represent the influenza types of primary concern for seasonal epidemics and pandemic preparedness. Specifically, the aims of this study are to (i) quantify the relative contribution of sequential versus overlapping infection pathways to COVID-19 and influenza transmissions; (ii) attribute new infections to their source class (singly-infected, co-infected, or sequentially-infected individuals); and (iii) evaluate how vaccination and non-pharmaceutical interventions (NPIs) differentially affect these transmission pathways.

## 2. Materials and Methods

### 2.1. The Co-Infection Model

We developed a deterministic compartmental model that couples a Susceptible–Vaccinated–Infected–Recovered (SVIR) model for influenza with a Susceptible–Vaccinated–Exposed–Infected (Asymptomatic/Symptomatic)–Hospitalized–Recovered (SVEAIHR) model for COVID-19 (note that in the descriptions that follow we have used disease and its causative agent (influenza and influenza virus, or COVID-19 and SARS-CoV-2) interchangeably). The resulting SVIR−SVEAIHR model serves as our primary framework to describe the distinct epidemiological states that arise during the co-circulation. Conceptually, the model can be interpreted as tracking a population through three parallel lanes of infections: a COVID-19-only lane, a concurrent co-infection lane, and an influenza-only lane, with cross-connections representing sequential infections. The model assumes a stable, homogeneously mixing population (i.e., every individual has an equal probability of contacting any other individual), which is a standard simplifying assumption in deterministic epidemic models. We acknowledge that this assumption may limit the ability to capture heterogeneity underlying real-world contact patterns due to factors such as age structure, geographic variation, or behavioral differences; however, it provides a tractable framework for isolating the mechanistic effects of infection history on transmission dynamics. The model allows for waning of both natural and vaccine-induced immunity. The model structure also allows for the inclusion of non-pharmaceutical interventions (NPIs) and vaccination, which are analyzed as intervention scenarios.

**Figure 1:**
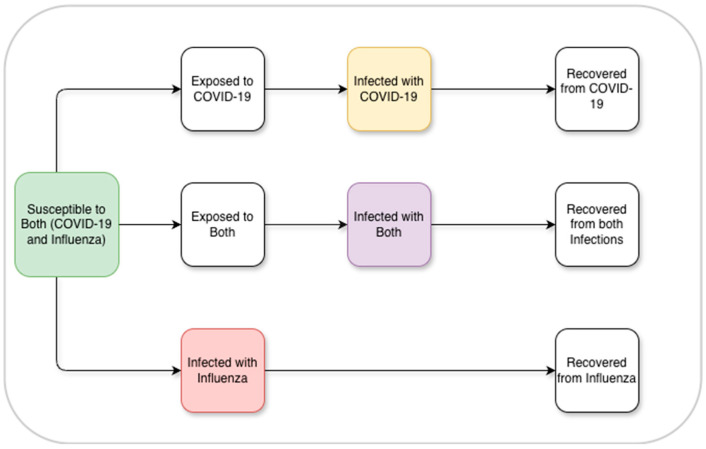
Flow diagram of the COVID-19–influenza co-infection model. A single-source susceptible compartment feeds three branches of transmission pathways: COVID-19 (**top**), concurrent COVID-19-influenza co-infection (**middle**), and influenza (**bottom**). Within each branch, only the core transitions (exposure/infection and recovery) are shown. Cross-transmission pathways (e.g., exposure/infection by one virus after recovery from the other) are not displayed here for clarity. Infectious compartments are color-coded: influenza-only (red), COVID-19-only (light yellow), and co-infectious (purple). A detailed version of the flow diagram is provided in the Supplementary Information (Figure S1), which includes the full model schema and parameterization.

Figure 1 is a simplified representation of our co-infection model. The complete model specification, including the system of ordinary differential equations (System S1) and an extended *SVEIR–SVEAIHR* variant (System S2), is provided in Supplementary Materials (S1.1 and S1.2).

### 2.2. The Model Outcomes

The primary outcomes were *Cumulative Incidence*, defined as the total fraction of the population newly infected over the 180-day simulation, and *Disease Burden*, defined as the total person-days of illness over the analysis horizon. As a secondary outcome, we also measured *Prevalence*, which is the fraction of the population actively infectious at a given time, to visualize the epidemic peaks (see Supplementary Section S2 for the full formulation of these model outcomes).

We decompose incidence and burden using two classifications:

1. Transmission Pathway: This classifies *how* an individual becomes infected. Sequential infection occurs when an infection follows the recovery from another infection (e.g., an influenza-recovered person gets infected with SARS-CoV-2). Overlapping (or concurrent) infections occur when a second virus is acquired while the first infection is still active.
2. Transmission Source: This attributes new infections to the status of the infectious population. We distinguish infections *attributable to* three source classes: singly-infected (individuals infectious with only one virus), co-infected (individuals infectious with multiple (typically two) viruses), and sequentially-infected (individuals infected with a virus after having recovered from another infection).

Within this framework, we also decompose COVID-19 transmission and burden by clinical presentation (Asymptomatic, Symptomatic, and Hospitalized) to analyze their respective contributions. This component directly supports the main objective of understanding co-infection dynamics. As the sequential pathway is the dominant driver of COVID-19 transmission, identifying the clinical subgroup (asymptomatic or symptomatic) responsible for that transmission may have direct implications for detection and control. Asymptomatic individuals, for instance, are harder to identify and isolate, meaning a sequential epidemic fueled largely by undetected cases would be especially difficult to interrupt. The clinical presentation decomposition, therefore, adds an actionable layer to the source–pathway analysis.

We parameterized the model using published parameter values from the epidemiological literature on influenza and COVID-19, with full details (definitions, baseline values, units, sources, and initial conditions for all 20 state variables) provided in Supplementary Tables S1 and S2.

For the baseline, our simulation assumes no NPIs, no vaccination and cross-susceptibility set to neutral (i.e., a prior infection with one virus does not alter the susceptibility to the other, providing a conservative lower bound on cross-viral interaction effects). Also, the transmission rates for both pathogens were calibrated to yield a basic reproduction number ($R_{0}$) of approximately 2.0 to facilitate an equitable comparison. It is important to note that $R_{0}$ = 2.0 serves as an invasion threshold and does not imply that a co-infected case generates two new co-infections. This equal-*R*_0_ baseline was chosen deliberately to isolate the effects of infection history and pathway structure from differences in intrinsic transmissibility; scenarios with unequal *R*_0_ values are explored in the sensitivity analysis (Section 2.4 and Table 1).

### 2.3. Intervention Scenarios

We examined how interventions influence the joint dynamics of influenza and COVID-19. We modeled two types of intervention scenarios. First, pathogen-specific vaccination was implemented by reducing the susceptibility of vaccinated individuals, with the model accounting for: (i) population coverage via a daily rollout rate (ranging from 0.5% to 1.5% of the susceptible population per day); (ii) vaccine effectiveness, modeled as the proportional reduction in susceptibility upon vaccination (parameterized from published estimates for both COVID-19 mRNA vaccines and seasonal influenza vaccines; see Supplementary Table S1); and (iii) the rate of waning immunity, which determines the duration of vaccine-induced protection. Note that the vaccination model does not differentiate between specific vaccine platforms (e.g., mRNA vs. inactivated), nor does it account for dosing schedules, as our aim was to assess the impact of population-level coverage and effectiveness rather than product-specific dynamics. Second, NPIs, representing community-level measures such as social distancing, masking, and reduced indoor gatherings, were modeled as a proportional reduction to the effective contact rate. This approach represents a composite NPI effect rather than modeling any single measure, which is appropriate for population-level epidemic modeling where individual NPI types are difficult to disentangle.

### 2.4. Sensitivity Analysis

We performed three targeted analyses to assess the robustness of our results to changes in virus introduction timing, model structure, and pathogen transmissibility.

First, to simulate different co-circulation scenarios, we fixed all parameters and $R_{0}$ calibration and varied only the relative introduction (seeding) times of the two pathogens. We simulated scenarios with 0, 30, and 60–days introduction delays under two distinct stagings: “flu-first” (influenza seeded before COVID-19) and “COVID-first” (COVID-19 seeded before influenza).

Second, to test structural parsimony, we compared outcomes from our primary *SVIR*-based model (which assumes no latent period for influenza) against a more complex *SVEIR*-based model (which includes a latent period; see Supplementary Figure S2 for the model flow diagram). This comparison assessed whether the exclusion of the exposed compartment for influenza materially affected the observed co-epidemic dynamics.

Third, we examined the impact of relative transmissibility by simulating scenarios with three distinct values for $R_{0}$ pairs (COVID-19 $R_{0c}$ and influenza $R_{0f}$): (1) higher influenza transmissibility ($R_{0c}=1.5,R_{0f}=2.0$); (2) equal baseline transmissibility ($R_{0c}=2.0,R_{0f}=2.0$); and (3) higher COVID-19 transmissibility ($R_{0c}=2.0,R_{0f}=1.5$).

Finally, we also explored the global sensitivity analysis (see Supplementary Section S3.3) and the impact of potential biological interactions by modifying the cross-infection susceptibility parameters (see Supplementary Section S5.4).

## 3. Results

### 3.1. Baseline Co-Epidemic Dynamics

**Figure 2:**
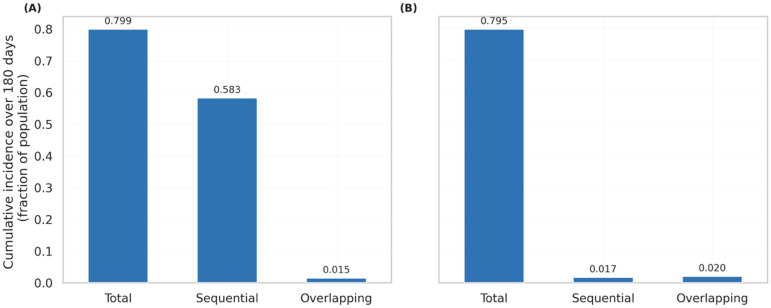
Cumulative incidence by transmission pathway for COVID-19 (**A**) and Influenza (**B**) (baseline). “Cumulative incidence” is the fraction of the population infected over 180 days. “Sequential” refers to infection from a virus after recovery from a different viral infection. “Overlapping” refers to a viral infection acquired while a different viral infection is still active, that is, two different viral infections overlapping for a certain period. Baseline simulation assumes no NPIs, no vaccination, and cross-susceptibility set to neutral.

Our analysis of the primary SVIR-based model (under baseline conditions) reveals a marked asymmetry in how the two viruses propagate when co-circulating. The pathway-specific incidence (Figure 2) shows that for COVID-19, the sequential pathway is dominant: infections among individuals who have recently recovered from influenza account for a cumulative incidence (fraction of the population infected over 180 days) of 0.583, i.e., ~73% of the total COVID-19 incidence (0.799). By contrast, the influenza epidemic is not primarily driven by co-infection dynamics: the sequential (0.017) and overlapping (0.020) pathways together contribute only a small fraction of total influenza incidence (0.795), implying that influenza cases arise mainly from direct transmission between singly infected and susceptible hosts (~95%).

**Figure 3:**
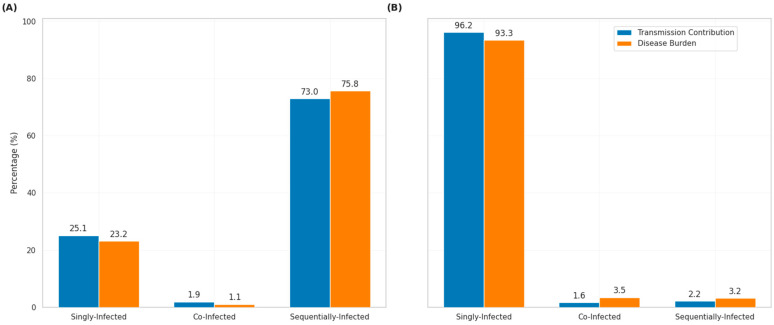
Transmission contribution versus disease burden by infection source (baseline). Bars show the percentage of total new infections (Transmission Contribution) and total person-days of illness (Disease Burden) attributable to each source class (Singly-infected, Co-infected, Sequentially-infected) for (**A**) COVID-19, and (**B**) Influenza. “Singly-infected” refers to individuals infected with only one type of virus. “Co-infected” refers to individuals infected with different viruses simultaneously (influenza virus and SARS-CoV-2 here). “Sequentially-infected” refers to individuals contracting a viral infection after having recovered from a different viral infection (contracting SARS-CoV-2 after influenza recovery (**A**), or contracting influenza virus after COVID-19 recovery (**B**)). Baseline simulation assumes no NPIs, no vaccination, and cross-susceptibility set to neutral.

Our source–decomposition analysis (Figure 3) further clarifies this asymmetry. For COVID-19, most new infections (approximately 73.0% in the baseline model) and the majority of the disease burden (approximately 75.8%) originate from the sequentially infected source group. Conversely, influenza transmission is overwhelmingly generated by non-sequential, only influenza virus infected individuals (approximately 96.2%, “Singly-infected” in Figure 3B) in the co-infection epidemic scenario, who also accrue most of the influenza burden (approximately 93.3%). It should be noted that these percentages represent model-based estimates under the specific baseline assumptions (equal *R*_0_ = 2.0, neutral cross-susceptibility, no NPIs); the estimates may vary with alternative parameter values, as confirmed by the sensitivity analysis (Table 1). Co-infected individuals contribute minimally to the forward spread for either virus in this baseline scenario. This low contribution from the co-infected class is mechanistically expected: concurrent co-infections require simultaneous susceptibility to both viruses, making them a relatively rare event in the model. Furthermore, the short infectious period limits the time window during which a co-infected individual can transmit both viruses, and therefore, even if some enhancement of transmissibility occurs, the small size of this population limits its contribution to the overall transmission.

**Figure 4:**
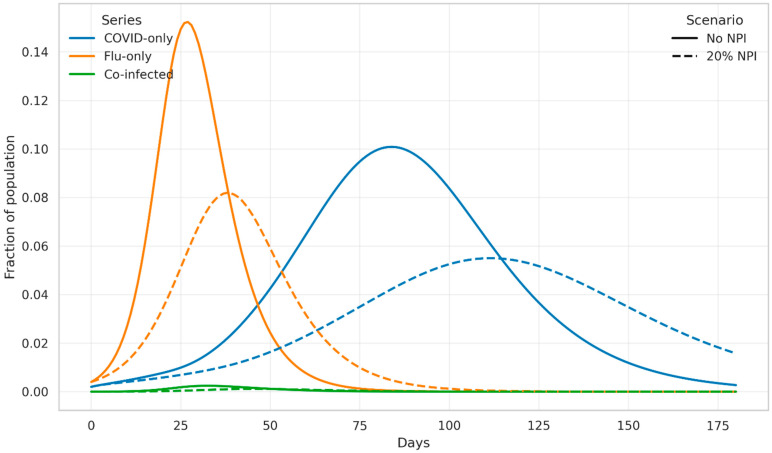
Infection prevalence of COVID-only, influenza-only, and COVID-influenza co-infected populations (baseline). Prevalence (fraction of the total population) is shown over 180 days for two scenarios: no interventions (solid lines) and a 20% reduction in effective contacts via NPIs (dashed lines). Baseline simulation assumes no NPIs, no vaccination and cross-susceptibility set to neutral.

Figure 4 illustrates the temporal dynamics of the epidemic. The baseline simulation resulted in two distinct waves. The first, driven by influenza, reaching a peak prevalence of approximately 15.2% of the population around day 27. A second, later wave is driven by COVID-19, which peaks around day 84 with a maximum prevalence of about 10%.

Furthermore, our analysis of the COVID-19 clinical presentation shows that asymptomatic individuals are the primary drivers of COVID-19 transmission (48.9%), while symptomatic individuals account for the majority of the disease burden (60.6%) (Supplementary Figure S6).

### 3.2. Impact of Interventions

**Figure 5:**
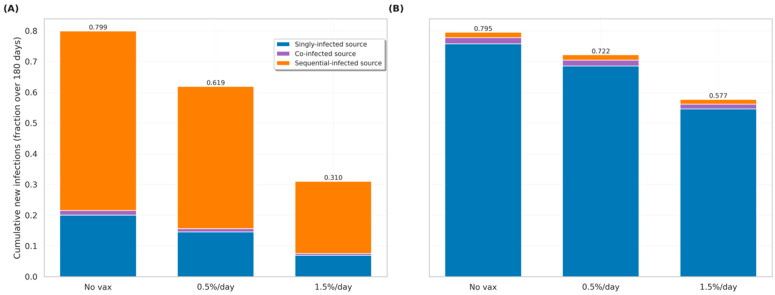
Cumulative incidence by source class under increasing vaccination rollout. Total cumulative infections (in terms of fraction of population over 180 days) for COVID-19 (**A**) and influenza (**B**) are shown for three vaccination scenarios: No vaccine (No Vax), a 0.5% per day rollout, and a 1.5% per day rollout. Bars are apportioned and color-coded by transmission sources: Singly-infected (individuals infectious with only one type of virus- shown in blue for COVID-19 (**A**) and influenza (**B**)), co-infected (individuals infectious with both viruses, shown in purple), and sequentially-infected (individuals contracting a viral infection after having recovered from a different viral infection- shown in orange for COVID-19 following recovery from influence (**A**) and for influenza after recovery from COVID-19 (**B**)).

Figure 5 illustrates the cumulative incidence under different vaccination scenarios. For COVID-19, where sequential transmission dominates, cumulative incidence declined from 0.799 without vaccination to 0.619 with a 0.5% per day rollout and to 0.310 with a 1.5% per day rollout. Reductions were greatest for the infections seeded by the sequentially infected source population. For influenza, where transmission is driven almost entirely by singly infected sources, vaccination still reduced total incidence from 0.795 to 0.577 at the 1.5% per day rate but had little effect on the already-minimal sequential or co-infected route.

**Figure 6:**
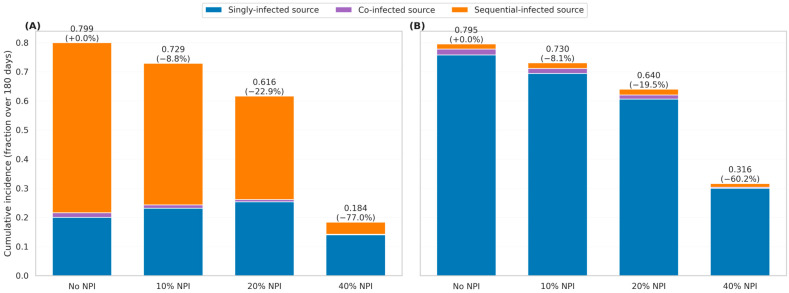
Cumulative incidence by source class under increasing NPIs. Total cumulative infections (in terms of fraction of population over 180 days) for COVID-19 (**A**) and influenza (**B**) are shown for four NPI scenarios: No NPI (0% effective contact reduction), 10% NPI (10% reduction), 20% NPI (20% reduction), and 40% NPI (40% reduction). Bars are apportioned and color-coded by transmission sources: Singly-infected (individuals infectious with only one type of virus- shown in blue for COVID-19 (**A**) and influenza (**B**)), co-infected (individuals infectious with both viruses, shown in purple), and sequentially-infected (individuals contracting a viral infection after having recovered from a different viral infection—shown in orange for COVID-19 following recovery from influence (**A**) and for influenza after recovery from COVID-19 (**B**)).

Figure 4 (dashed lines) illustrates the epidemic response to NPIs. An NPI reducing effective contacts by 20% flattens and delays the peaks of both COVID-19 and influenza epidemics. This NPI reduces the peak prevalence by approximately 46% for influenza (to 0.082 at day 38) and approximately 45.5% for COVID-19 (to 0.055 at day 111), while delaying the peaks by 11–27 days. The impact on total incidence is also substantial as the NPI is increased up to 40% and thus reducing the effective contacts by up to 40% (Figure 6). The COVID-19 cumulative incidence fell from 0.799 (no NPI) to 0.184 at a 40% reduction in effective contacts. While absolute infections from all sources fell, the relative contribution from singly infected sources became larger at modest NPI strengths (Figure 6). For influenza, NPIs primarily reduced infections generated by the dominant singly (influenza only) infected sources, with cumulative incidence falling from 0.795 to 0.316 at 40% reduction in effective contacts; contributions from other sources remained negligible.

Combining NPI and vaccination yielded the lowest joint burden (Supplementary Figure S8). Relative to the baseline (COVID-19: 0.799; influenza: 0.795), NPIs alone (at 20%) produced moderate declines (COVID-19: 0.618; influenza: 0.640). Adding a COVID-19 vaccine on top of NPIs sharply reduced COVID-19 incidence to 0.311 with little change in influenza. Conversely, adding an influenza vaccine on top of NPIs primarily reduced the influenza incidence. Deploying both vaccines alongside NPIs resulted in the smallest combined footprint (COVID-19: 0.335; influenza: 0.527).

### 3.3. Sensitivity Analysis Outcomes

**Impact of Relative Introduction Timing:** In the “flu-first” scenario (Supplementary Figure S7A), the contribution of the sequential pathway to COVID-19 infections ranged from 72.9% to 78.6%. At a 0-day lag, the sequential pathway accounted for 72.9% of infections. This proportion increased to 78.3% at a 30-day lag and 78.6% at a 60-day lag. The overlapping pathway contributed 1.9% at a 0-day lag and declined to nearly zero as the delay increased.

In the “COVID-first” scenario (Supplementary Figure S7B), the contribution of the sequential pathway to influenza infections increased with the length of the delay. At a 0-day lag, the influenza transmission was 95.2% attributable to the singly-infected pathway. As the delay increased to 30 and 60 days, the sequential share of influenza infections increased to 12.4% and 38.3%, respectively. The overlapping pathway shares for influenza increased from 2.6% at a 0-day lag to 14.6% at a 60-day lag.

**Impact of Influenza Model Structure:** Comparison of the primary *SVIR* model (no latent period) with the *SVEIR* model (with latent period) showed differences in source contributions. In the *SVEIR* model, the influenza epidemic peak was lower and occurred later compared to the *SVIR* model (Supplementary Figure S4). The contribution of the sequential pathway to COVID-19 incidence was 68.2% in the *SVEIR* model, compared to 73.4% in the primary *SVIR* model (Table 1). Influenza transmission remained driven by the singly-infected pathway in both frameworks, albeit relatively less pronounced with *SVEIR* (91.3% in *SVEIR* vs. 96.0% in *SVIR*).

**Impact of Pathogen Transmissibility:** We observed changes in the sequential share of transmission under varying transmissibility scenarios (Table 1). In the scenario with higher influenza transmissibility ($R_{0c}=1.5,R_{0f}=2.0$), the sequential pathway accounted for 75.5% of COVID-19 transmission and 76.6% of the disease burden. In the scenario with higher COVID-19 transmissibility ($R_{0c}=2.0,R_{0f}=1.5$), the sequential share was 46.2% for transmission and 50.9% for burden. Across all considered scenarios (Table 1; see also Supplementary Figure S3 for the full source-decomposition bar charts under each *R*_0_ pair), the singly-infected pathway accounted for 75.2% to 97.9% of total influenza transmission.

Taken together, the key takeaway from Table 1 is threefold. First, the dominance of the sequential pathway for COVID-19 transmission is a robust finding: across all six model-structure and transmissibility combinations tested, the sequential source accounts for the majority of COVID-19 incidence (range: 37.1–75.5%) and burden, with the dominance being strongest when influenza is more transmissible. Second, the influenza transmission is consistently driven by the singly-infected class regardless of model structure or transmissibility, confirming that influenza largely behaves as a single-pathogen epidemic in these co-circulation settings. Third, the magnitude of the sequential COVID-19 share is sensitive to the relative transmissibility of the two viruses: when COVID-19 is more transmissible than influenza (*R*_0*c*_ = 2.0, *R*_0*f*_ = 1.5), the sequential share drops substantially (37.1–46.2%), indicating that the asymmetry depends on the scale of the initial influenza wave.

**Global Sensitivity:** The global sensitivity analysis (Supplementary Section S4.3, Table S3; PRCC tornado plots in Supplementary Figure S5) indicated that the cross-susceptibility parameter was positively correlated with total COVID-19 incidence (Supplementary Figure S9).

## 4. Discussion

**The Asymmetry of Co-Epidemic Dynamics.** This study’s central contribution is the demonstration that infection history is a primary and asymmetric driver of co-epidemic dynamics. By decomposing transmission into sources and pathways—a methodological advance over conventional aggregated models—we revealed that an initial influenza wave can fundamentally reshape the transmission landscape for a subsequent COVID-19 epidemic. This occurs because the co-circulation of these viruses generates an epidemic with a significantly different timing compared to independent outbreaks. This temporal staging of the two viruses generates a large, transient reservoir of influenza-recovered individuals, which then serves as the primary engine for COVID-19 spread. Based on our model prediction, the sequential pathway is estimated to account for approximately 73% of all COVID-19 cases and 76% of the disease burden, a non-obvious finding that challenges the notion that single-pathogen dynamics are sufficient to understand co-circulation, as the majority of COVID-19 transmission in this scenario occurred through a pathway that does not exist in a single-pathogen model. Contextualizing this finding relative to prior modeling work is instructive. Previous COVID-19–influenza co-infection models, such as those by Liang et al. [7], Ojo et al. [4], and Bhowmick et al. [5], have shown that co-circulating pathogens can substantially elevate joint disease burden and that combined interventions outperform single-pathogen strategies. While our work complements these findings, it extends them in a critical direction—while those studies analyzed total burden and intervention effectiveness, they treated the co-infected population as a single aggregate class; in contrast, our decomposition framework reveals that the transmission dynamics within that class are highly heterogeneous and that the sequential pathway (post-influenza COVID-19) is the dominant driver—a distinction that cannot be discerned by conventional aggregated models. McKinney et al. [8] similarly found that hospitalization dynamics are sensitive to the interaction between two pathogens they considered, consistent with our finding that sequential infection substantially elevates the disease burden.

**Mechanistic Explanations for Clinical Observations.** Our population-level model provides a mechanistic framework for interpreting conflicting real-world observations. While some epidemiological studies suggest prior influenza may increase COVID-19 severity [11,14], others have found evidence of viral interference, where influenza infection reduces the odds of testing positive for SARS-CoV-2 [15,16]. Our results suggest these are not mutually exclusive. It is important to distinguish, however, what our model infers and what still remains unresolved, framed as a working hypothesis. Our model predicts that if influenza infection increases susceptibility to SARS-CoV-2 (even moderately), the sequential transmission pathway becomes dominant. As a working hypothesis that our model appears consistent with but does not prove, this sequential pathway may be biologically driven by in vitro evidence of enhanced SARS-CoV-2 infectivity post-influenza, potentially driven by biological mechanisms such as epithelial damage or ACE2 upregulation [2,3,17,18]. This suggests a scenario where influenza creates a transiently hyper-susceptible population [11], explaining the powerful transmission dynamic, even if direct co-infection is suppressed by short-term interference. Our model’s predictions are consistent with these reports suggesting potential biological mechanisms; however, they represent hypotheses requiring empirical confirmation in human populations; the model in itself cannot distinguish between different biological mechanisms underlying the cross-susceptibility effect.

**Robustness and Sensitivity.** The asymmetry in transmission sources was robust across changes in model structure and pathogen transmissibility. The comparison between the SVIR and SVEIR frameworks demonstrated that while the inclusion of a latent period altered the precise timing of the epidemic, the dominance of the sequential pathway for COVID-19 remained consistent. Furthermore, the sensitivity analysis revealed a clear dose-response relationship between the relative transmissibility of influenza and the magnitude of the sequential COVID-19 epidemic. A more transmissible influenza variant ${(R}_{0f}=2.0$) amplified the sequential effect compared to a scenario where COVID-19 was dominant ($R_{0c}=2.0$), confirming that the scale of the initial epidemic is a key determinant of the subsequent epidemic trajectories in co-infection scenarios.

**Pathway-Specific Intervention Effects.** Our decomposition approach offers a novel method for interpreting the impact of public health interventions. Other modeling work has shown that combined vaccination strategies are essential for mitigating severe outcomes [4,18,19] and that NPIs can delay and flatten co-epidemic peaks [7]. Our work adds a critical layer of granularity. The key insight is that interventions have distinct, pathway-specific effects: COVID-19 vaccination, for example, is disproportionately effective in a co-epidemic because it directly disrupts the dominant sequential transmission engine. This reveals a synergistic benefit to COVID-19 vaccination during influenza season. Conversely, our finding that moderate NPIs can paradoxically increase the relative importance of the singly-infected source, by preserving a larger pool of fully susceptible individuals (an observation reported in [20]), highlights the complex, and sometimes counterintuitive, ways that broad control measures reshape transmission dynamics. Together, these results argue for a shift towards pathway-aware public health strategies that consider not just the total number of infectious individuals, but also the epidemiological pathways that contribute to that pool.

**Public Health Implications.** The insights gained from this study can be translated into actionable public health intelligence. The findings generate concrete, testable hypotheses and strategic recommendations that reach beyond addressing separate epidemics; specifically, towards managing interconnected systems of multiple pathogens. The most urgent testable hypothesis is the need to empirically quantify the magnitude of post-influenza susceptibility to SARS-CoV-2 in human populations; our model’s high sensitivity to this parameter, as shown in our global sensitivity analysis, highlights this as a critical knowledge gap. Strategically, public health planning should consider “epidemic sequencing”. For example, following a severe influenza season, health systems could anticipate a potential surge in COVID-19 driven by the sequential pathway and proactively ramp up testing, wastewater surveillance, and targeted vaccination campaigns for vulnerable populations. This would involve using influenza surveillance data—timing, size, and severity—as a direct input for short-term COVID-19 forecasting models, creating a novel, data-driven strategy for managing the combined threat of co-circulating respiratory viruses.

**Limitations and Practical implications.** It is crucial, however, to interpret our findings within the context of the model’s assumptions, such as homogeneous mixing, and the parameterization aimed at comparability. While the precise percentages will vary with alternative assumptions (e.g., partial cross-immunity or age structure), the qualitative conclusions are robust across the tested frameworks: infection sequence matters, COVID-19 can be strongly fueled by post-influenza hosts, and influenza largely mirrors a single-pathogen (influenza only) dynamic in these co-circulation settings. A note regarding real-world implementation: the model does not incorporate country-specific demographic data, age structure, or gender differences in susceptibility or outcomes. These factors would be important for adapting the framework to specific epidemiological contexts, and we consider their inclusion a key direction for future model extensions. The practical implications of the current results are nevertheless profound. First, epidemic sequencing should inform preparedness: a strong, early influenza season should trigger heightened readiness for a subsequent COVID-19 surge, including proactive COVID-19 vaccination campaigns, expanded wastewater and sentinel surveillance, and targeted outreach to populations most likely to be sequentially exposed. Second, the evaluation of interventions should be pathway-aware, recognizing that total case numbers can conceal important shifts in transmission sources. Third, the high sensitivity of outcomes to cross-susceptibility parameters underscores a critical data need: the empirical quantification of post-influenza susceptibility to COVID-19 to refine future forecasting models. In summary, by using a decomposition framework to attribute new infections to their source and pathway, this study reveals a robust, sequence-driven asymmetry that offers a clear, evidence-based route to more effective, layered mitigation of co-circulating respiratory viruses.

## 5. Conclusions

Our work reveals the importance of infection history in understanding the dynamics of viral co-epidemics, based on a mechanistic modeling framework. Our model-based findings suggest that the interventions may have pathogen- and pathway-specific effects, and support the value of layering control strategies for managing co-circulating respiratory viruses. These conclusions, however, must be cautiously interpreted in light of the model’s simplifying assumptions, including homogeneous mixing and parameterization from the published estimates rather than from the real-world surveillance data. Future research directions emerging from this work may include: (i) fitting the decomposition framework to the real-world co-epidemic surveillance data to empirically validate the dominance of the sequential pathway; (ii) extending the model to incorporate age structure and heterogeneous contact patterns; (iii) empirically quantifying the magnitude of post-influenza susceptibility to SARS-CoV-2 in human populations, which our global sensitivity analysis identifies as the most critical parameter; and (iv) exploring how emerging influenza variants or new SARS-CoV-2 lineages with altered cross-susceptibility profiles might shift the balance of transmission pathways. Considering these directions could be crucial for translating the pathway-aware insights from this model into actionable, evidence-based public health guidance.

## Figures and Tables

**Table 1:** Summary of Sensitivity Analyses on Transmission Sources.

Model Structure	$R_{0}$ Pair ${(R}_{0c},\;R_{0f})$	COVID Sequential Share (Transmission)	COVID Sequential Share (Burden)	Flu Singly-Infected Share (Transmission)	Flu Singly-Infected Share (Burden)
**SVIR (Primary)**	**(2.0, 2.0)**	**73.4%**	**76.1%**	**96.0%**	**93.4%**
**SVIR (Primary)**	(1.5, 2.0)	75.5%	76.6%	97.9%	96.9%
**SVIR (Primary)**	(2.0, 1.5)	46.2%	50.9%	87.6%	82.7%
**SVEIR (Sensitivity)**	**(2.0, 2.0)**	**68.2%**	**72.9%**	**91.3%**	**87.7%**
**SVEIR (Sensitivity)**	(1.5, 2.0)	73.1%	74.7%	96.2%	95.2%
**SVEIR (Sensitivity)**	(2.0, 1.5)	37.1%	43.8%	75.2%	70.1%

Note: $R_{0c}$ and $R_{0f}$ denote the basic reproduction numbers for COVID-19 and Influenza, respectively. “*SVIR* (Primary)” refers to the baseline model which assumes no latent period for influenza, while “*SVEIR* (Sensitivity)” refers to the structural sensitivity model that includes a latent (Exposed) period. “Sequential Share” represents the percentage of new infections occurring in individuals who have previously recovered from the other virus. Rows shown bold-faced display the corresponding results for the two model structures under equal $R_{0}$ values.

## Data Availability

The code used to generate all model simulations and figures in this study is available from the corresponding author upon reasonable request. All model parameters, initial conditions, and equation systems required to reproduce the results are provided in Supplementary Tables S1 and S2 and Supplementary Section S1.

## Supplementary Materials

The following supporting information can be downloaded at: https://www.mdpi.com/article/10.3390/microorganisms14061239/s1. Figure S1: Flow diagram of the primary *SVIR–SVEAIHR* co-infection model. The model tracks population movement based on influenza status (vertical flow, SVIR structure) and COVID-19 status (horizontal flow, *SVEAIHR* structure). Compartments are color-coded by disease infectious status: influenza-only (red), COVID-19-only (light yellow), and co-infectious (purple). Dashed boxes represent waning immunity returning individuals to a susceptible state; Figure S2: Flow diagram of the extended *SVEIR–SVEAIHR* co-infection model (sensitivity model). This model is structurally identical to the primary model but includes an explicit latent (Exposed) period for influenza (the X_Ef_j compartments), adding 6 new compartments for a total of 26; Figure S3: Transmission contribution and disease burden by infection source under different pairs of basic reproduction numbers (*R*_0_) using the *SVIR–SVEAIHR* model. Panels A–C show results for (*R*_0*c*_ = 2.0, *R*_0*f*_ = 2.0), (*R*_0*c*_ = 2.0, *R*_0*f*_ = 1.5), and (*R*_0*c*_ = 1.5, *R*_0*f*_ = 2.0), respectively. First column: COVID-19; second column: influenza; Figure S4: Comparison of epidemic dynamics using *SVIR* vs. *SVEIR* influenza model structures under different *R*_0_ pairs. Curves show prevalence over time for influenza-only, COVID-only, and co-infected populations. Panels A–C: *SVIR–SVEAIHR*; Panels D–F: *SVEIR–SVEAIHR*; Figure S5: Cumulative incidence partial rank correlation coefficient (PRCC) tornado plots with 95% bootstrap confidence intervals. Bars to the right (blue) indicate parameters that increase incidence; bars to the left (red) indicate parameters that decrease incidence. Panel (a): COVID-19; Panel (b): influenza; Figure S6: Comparison of contribution to total COVID-19 transmission versus disease burden, broken down by clinical presentation (Asymptomatic, Symptomatic, and Hospitalized). Bars are normalized to 100% and integrated over 180 days; Figure S7: Pathway shares versus inter-epidemic delay. Share of cumulative incidence by pathway (direct, sequential, overlapping) when one epidemic precedes the other by 0, 30, or 60 days. Panel A: flu-first (showing pathways for COVID-19); Panel B: COVID-first (showing pathways for influenza); Figure S8: Cumulative incidence (fraction of population infected over 180 days) under five combined intervention strategies: baseline (no interventions), NPI only (20% reduction in effective contacts), NPI + COVID-19 vaccination, NPI + influenza vaccination, and NPI + both vaccines; Figure S9: Sensitivity of co-epidemic outcomes to cross-susceptibility parameters ψc and ψf, varied from 0.6 to 1.6. Panel A: peak co-infection prevalence; Panel B: cumulative co-infection incidence; Panel C: share of total COVID-19 incidence from the sequential pathway (%); Panel D: share of total influenza incidence from the sequential pathway (%). The red dot marks the baseline (ψc = 1.0, ψf = 1.0); Table S1: Model parameter definitions, baseline values, units, and sources for all parameters; Table S2: Initial conditions for all 20 state variables with baseline seeded values; Table S3: Global sensitivity of cumulative incidence (180 days) to model parameters: Latin hypercube sampling–PRCC with 95% confidence intervals for COVID-19 and influenza outcomes; Section S1: Model equations and parameterization; Section S2: Mathematical formulation of incidence decomposition and disease burden accounting; Section S3: Sensitivity analysis methods (model structure, transmissibility, and global sensitivity); Section S4: Key findings from sensitivity analyses; Section S5: Additional numerical results. References [1,4,6,10,21,22,23,24,25,26,27,28,29,30] are cited in the Supplementary Materials.
