# Supplementary material for: Infection History Shapes Co-Epidemic Dynamics: A Transmission Source–Pathway Decomposition for COVID-19 and Influenza
Source: Microorganisms. 2026 May 30;14(6):1239. doi: 10.3390/microorganisms14061239 (PMC13302906; doi:10.3390/microorganisms14061239)
Supplement: Supplementary file 1 [file microorganisms-14-01239-s001.zip › microorganisms-4272440-supplementary.pdf]

Supplement for:

Infection History Shapes Co-Epidemic Dynamics: A Transmission Source–Pathway Decomposition for COVID-19 and Influenza

Mani Dhakal[1], Brajendra K. Singh[2], and Rajeev K. Azad[3]

[1]Department of Mathematics, University of North Texas, Denton, TX 76203, USA

[2]The Preserve at Killian Hill, Lilburn, Georgia, 30047, USA

[3]Department of Biological Sciences and BioDiscovery Institute, University of North Texas, Denton, TX 76203, USA

Table of Contents

S1. Model Equations, Parameters, and Initial Conditions 3
S1.1. The Primary Model 3
S1.2. The Sensitivity (Extended) model 6
S1.3. Model Parameterization 10
S1.4. Parameter values and Initial Conditions 10

S2. Mathematical Formulation of Incidence decomposition and Burden Accounting....... 12
S2.1 Pathway-specific incidence (per pathogen). 13
S2.2 Source-decomposed incidence. 13
S2.3 Disease burden 14
S2.4. Interventions 14
S2.5. Numerical Integration and units 15

S3. Sensitivity to Model Framework and Transmissibility (The Methods) 15
S3.1 Model Structure Sensitivity (SVIR vs. SVEIR for Influenza) 15
S3.2 Relative Transmissibility Sensitivity ($R0$ Pair Analysis) 15
S3.3 Global Sensitivity Analysis 16

S4. Key Findings from Sensitivity Analysis (The Results) 16
S4.1 Findings from Model Structure Sensitivity 16
S4.2 Findings from Relative Transmissibility Sensitivity 16
S4.3 Finding from Global Sensitivity Analysis 19

S5. Additional Numerical Results 21
S5.1 Transmission vs. Disease Burdens by Clinical Presentation (COVID-19) 21
S5.2 Impact of Epidemic Timing Scenarios 22
S5.3 Effect of Combined Intervention Strategies 23
S5.4 Sensitivity to Cross-Susceptibility 24

S1. Model Equations, Parameters, and Initial Conditions

S1.1. The Primary Model

The deterministic compartmental model (primary) couples an *SVIR* structure for influenza with an *SVEAIHR* structure for COVID-19. The model consists of 20 mutually exclusive compartments. Following standard notation schemes [1–4], the model's compartments are denoted by X_{ij} , where the subscript $i \in S_f, V_f, I_f, R_f$ represents an individual's status with respect to influenza and the subscript $j \in S_c, V_c, E_c, A_c, I_c, H_c, R_c$ represents their status with respect to COVID-19. For example, $X_{I_f S_c}$ denotes the subpopulation currently infected with influenza but susceptible to COVID-19. The total population $N = \sum_{i,j} X_{ij}$ is assumed constant or near constant due to balancing birth (Λ) and death (μ_*) rates ($\Lambda = \mu N$ or $\mu N + disease\, related\, deaths$).The forces of infection for influenza (λ_f) and COVID-19 (λ_c) are defined underneath the equations. The model diagram is displayed in **Figure S1**.

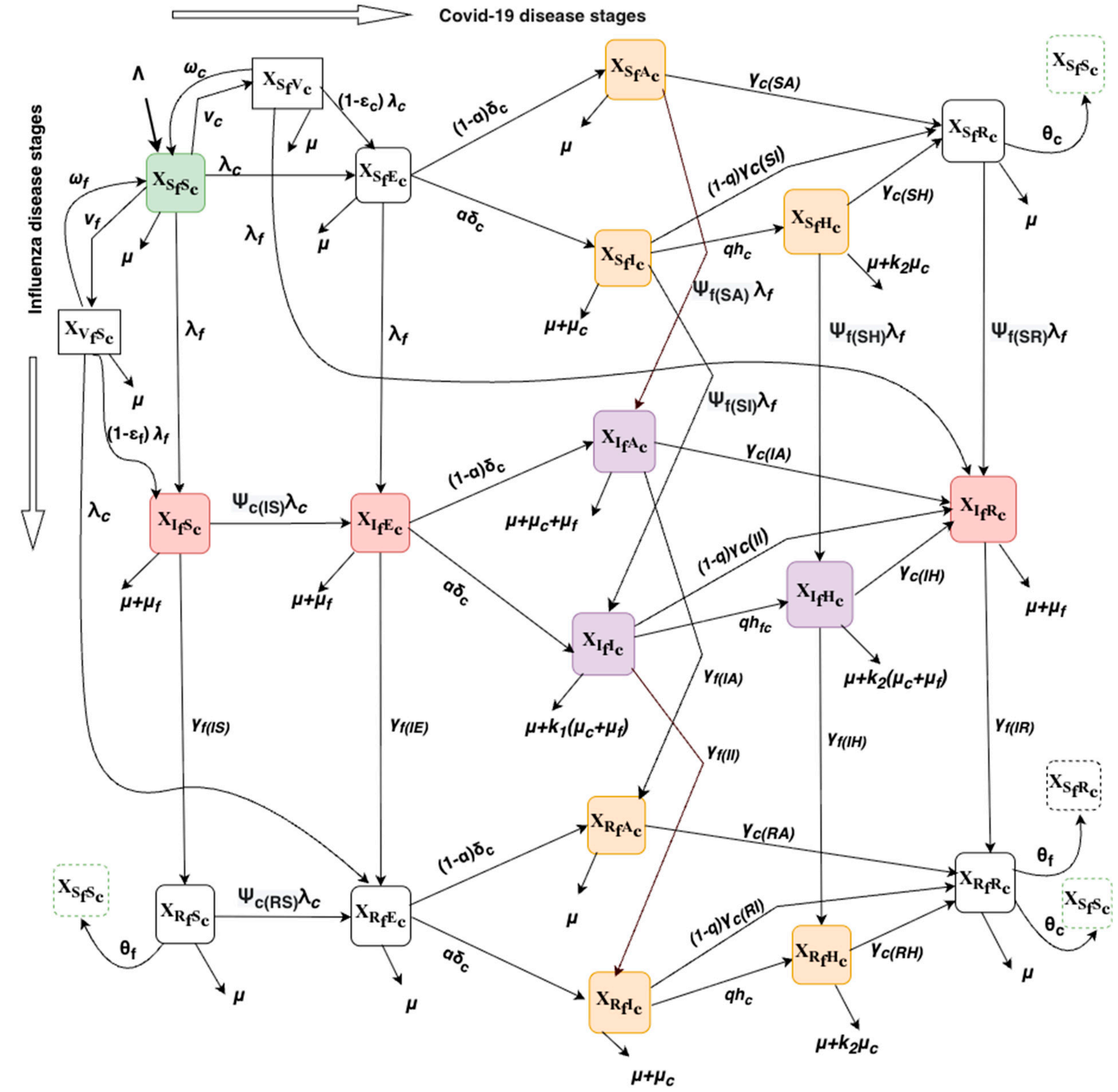

Figure S1: Flow diagram of the primary *SVIR-SVEAIHR* co-infection model**.** The model tracks population movement based on influenza status (vertical flow, SVIR structure) and COVID-19 status (horizontal flow, *SVEAIHR* structure). Compartments are color-coded by disease infectious status: influenza-only (red), COVID-19-only (light yellow), and co-infectious (purple). Dashed boxes represent waning immunity returning individuals to a susceptible state.

The full system of 20 ordinary differential equations (ODEs) corresponding to the model in **Figure S1**, is given below (we refer this set of equations as **System S1**):

$$\frac{dX_{S_fS_c}}{dt} = \Lambda + \theta_c(X_{S_fR_c} + X_{R_fR_c}) + \theta_f X_{R_fS_c} + \omega_c X_{S_fV_c} + \omega_f X_{V_fS_c} - \left(\frac{\beta_f I_F}{N} + \frac{\beta_c I_C}{N} + v_c + v_f + \mu\right) X_{S_fS_c}$$

$$\frac{dX_{S_fV_c}}{dt} = v_c X_{S_fS_c} - \left((1-\epsilon_c)\frac{\beta_c}{N} I_C + \frac{\beta_f}{N} I_F + w_c + \mu\right) X_{S_fV_c}$$

$$\frac{dX_{S_fE_c}}{dt} = (1-\epsilon_c)\frac{\beta_c}{N} I_C X_{S_fV_c} + \frac{\beta_c I_C X_{S_fS_c}}{N} - \left(\beta_f \frac{I_F}{N} + \delta_c + \mu\right) X_{S_fE_c}$$

$$\frac{dX_{S_fA_c}}{dt} = (1-\alpha_c)\delta_c X_{S_fE_c} - \left(\psi_{f(SA)}\beta_f \frac{I_F}{N} + \gamma_{c(A)} + \mu\right) X_{S_fA_c}$$

$$\frac{dX_{S_fI_c}}{dt} = \alpha_c \delta_c X_{S_fE_c} - \left(\psi_{f(SI)}\beta_f \frac{I_F}{N} + qh_c + (1-q)\gamma_{c(S)} + \mu + \mu_c\right) X_{S_fI_c}$$

$$\frac{dX_{S_fH_c}}{dt} = qh_c X_{S_fI_c} - \left(\psi_{f(SH)}\beta_f \frac{I_F}{N} + \mu + \kappa_2\mu_c + \gamma_{c(H)}\right) X_{S_fH_c}$$

$$\frac{dX_{S_fR_c}}{dt} = \gamma_{c(A)} X_{S_fA_c} + \theta_f X_{R_fR_c} + (1-q)\gamma_{c(S)} X_{S_fI_c} + \gamma_{c(H)} X_{S_fH_c} - \left(\psi_{f(SR)}\beta_f \frac{I_F}{N} + \mu + \theta_c\right) X_{S_fR_c}$$

$$\frac{dX_{V_fS_c}}{dt} = v_f X_{S_fS_c} - \left((1-\epsilon_f)\frac{\beta_f}{N} I_F + \frac{\beta_c}{N} I_C + w_f + \mu\right) X_{V_fS_c}$$

$$\frac{dX_{I_fS_c}}{dt} = (1-\epsilon_f)\frac{\beta_f I_F X_{V_fS_c}}{N} + \frac{\beta_f I_F X_{S_fS_c}}{N} - \left(\psi_{c(IS)}\beta_c \frac{I_C}{N} + \gamma_f + \mu + \mu_f\right) X_{I_fS_c}$$

$$\frac{dX_{I_fE_c}}{dt} = \psi_{c(IS)}\beta_c \frac{I_C X_{I_fS_c}}{N} + \beta_f \frac{I_F X_{S_fE_c}}{N} - (\delta_c + \gamma_f + \mu + \mu_f) X_{I_fE_c}$$

$$\frac{dX_{I_fA_c}}{dt} = (1-\alpha_c)\delta_c X_{I_fE_c} + \psi_{f(SA)}\beta_f \frac{I_F X_{S_fA_c}}{N} - (\zeta_c\gamma_{c(A)} + \zeta_f\gamma_f + \mu + \mu_c + \mu_f) X_{I_fA_c}$$

$$\frac{dX_{I_fI_c}}{dt} = \alpha_c\delta_c X_{I_fE_c} + \psi_{f(SI)}\beta_f \frac{I_F X_{S_fI_c}}{N} - \left(qh_{fc} + (1-q)\zeta_c\gamma_{c(S)} + \zeta_f\gamma_f + \mu + \kappa_1(\mu_c + \mu_f)\right) X_{I_fI_c}$$

$$\frac{dX_{I_fH_c}}{dt} = qh_{fc} X_{I_fI_c} + \psi_{f(SH)}\beta_f \frac{I_F X_{S_fH_c}}{N} - \left(\zeta_f\gamma_f + \mu + \kappa_2(\mu_c + \mu_f) + \zeta_c\gamma_{c(H)}\right) X_{I_fH_c}$$

$$\frac{dX_{I_fR_c}}{dt} = \frac{\beta_f I_F X_{S_fV_c}}{N} + \psi_{f(SR)}\beta_f \frac{I_F X_{S_fR_c}}{N} + \zeta_c\left(\gamma_{c(A)} X_{I_fA_c} + (1-q)\gamma_{c(S)} X_{I_fI_c} + \gamma_{c(H)} X_{I_fH_c}\right) - (\gamma_f + \mu + \mu_f) X_{I_fR_c}$$

$$\frac{dX_{R_fS_c}}{dt} = \gamma_f X_{I_fS_c} - \psi_{c(RS)}\left(\frac{\beta_c I_C}{N} + \mu + \theta_f\right) X_{R_fS_c}$$

$$\frac{dX_{R_fE_c}}{dt} = \beta_c \frac{I_C X_{V_fS_c}}{N} + \psi_{c(RS)}\beta_c \frac{I_C X_{R_fS_c}}{N} + \gamma_f X_{I_fE_c} - (\delta_c + \mu) X_{R_fE_c}$$

$$\frac{dX_{R_fA_c}}{dt} = (1-\alpha_c)\delta_c X_{R_fE_c} + \zeta_f\gamma_f X_{I_fA_c} - (\gamma_{c(A)} + \mu) X_{R_fA_c}$$

$$\frac{dX_{R_fI_c}}{dt} = \alpha_c\delta_c X_{R_fE_c} + \zeta_f\gamma_f X_{I_fI_c} - (qh_c + (1-q)\gamma_{c(S)} + \mu + \mu_c) X_{R_fI_c}$$

$$\frac{dX_{R_fH_c}}{dt} = qh_c X_{R_fI_c} + \zeta_f\gamma_f X_{I_fH_c} - (\mu + \kappa_2\mu_c + \gamma_{c(H)}) X_{R_fH_c}$$

$$\frac{dX_{R_fR_c}}{dt} = \gamma_{c(A)} X_{R_fA_c} + (1-q)\gamma_{c(S)} X_{R_fI_c} + \gamma_{c(H)} X_{R_fH_c} + \gamma_f X_{I_fR_c} - (\mu + \theta_c + \theta_f) X_{R_fR_c}$$

where, $I_F = X_{I_fS_c} + X_{I_fE_c} + X_{I_fA_c} + X_{I_fI_c} + \eta_{H_c} X_{I_fH_c} + X_{I_fR_c}$ and $I_C = \eta_{A_c} X_{S_fA_c} + \eta_{I_c} X_{S_fI_c} + \eta_{H_c} X_{S_fH_c} + \eta_{A_c} X_{I_fA_c} + \eta_{I_c} X_{I_fI_c} + \eta_{H_c} X_{I_fH_c} + \eta_{A_c} X_{R_fA_c} + \eta_{I_c} X_{R_fI_c} + \eta_{H_c} X_{R_fH_c}$ respectively are the total number of influenza infectious and COVID-19 infectious population at a particular instance of time (when infectious modification parameters $\eta_{A_c} = \eta_{I_c} = \eta_{H_c} = 1.0$. We note that the population in each compartment depends on time, and during a particular instance of time t we have, $N(t) = X_{S_fS_c}(t) + X_{S_fV_c}(t) + X_{S_fE_c}(t) + X_{S_fA_c}(t) + X_{S_fI_c}(t) + X_{S_fH_c}(t) + X_{S_fR_c}(t) + X_{V_fS_c}(t) + X_{I_fS_c}(t) + X_{I_fE_c}(t) + X_{I_fA_c}(t) + X_{I_fI_c}(t) + X_{I_fH_c}(t) +$

$X_{I_fR_c}(t) + X_{R_fS_c}(t) + X_{R_fE_c}(t) + X_{R_fA_c}(t) + X_{R_fI_c}(t) + X_{R_fH_c}(t) + X_{R_fR_c}(t)$. For the sake of notational simplicity, we drop t from the notation of the model variables in the descriptions that follow.

S1.2. The Sensitivity (Extended) model

The deterministic compartmental model (sensitivity) couples an ***SVEIR*** structure for influenza with the *SVEAIHR* structure for COVID-19. This model is an extension of the primary model (System S1) and includes an explicit latent (Exposed) period for influenza. This adds 6 new compartments ($\boldsymbol{X_{E_fS_c}}, \boldsymbol{X_{E_fE_c}}, \boldsymbol{X_{E_fA_c}}, \boldsymbol{X_{E_fI_c}}, \boldsymbol{X_{E_fH_c}}, \boldsymbol{X_{E_fR_c}}$) to the 20 compartments from System S1, for a total of 26 mutually exclusive compartments. The notation scheme ($\boldsymbol{X_{ij}}$) and definition of the total population ($\boldsymbol{N}$) are the same as described in Section S1.1.

The model diagram is displayed in **Figure S2** and the full system of 26 ODEs corresponding to the model in **Figure S2** is given below (we refer this as **System S2**):

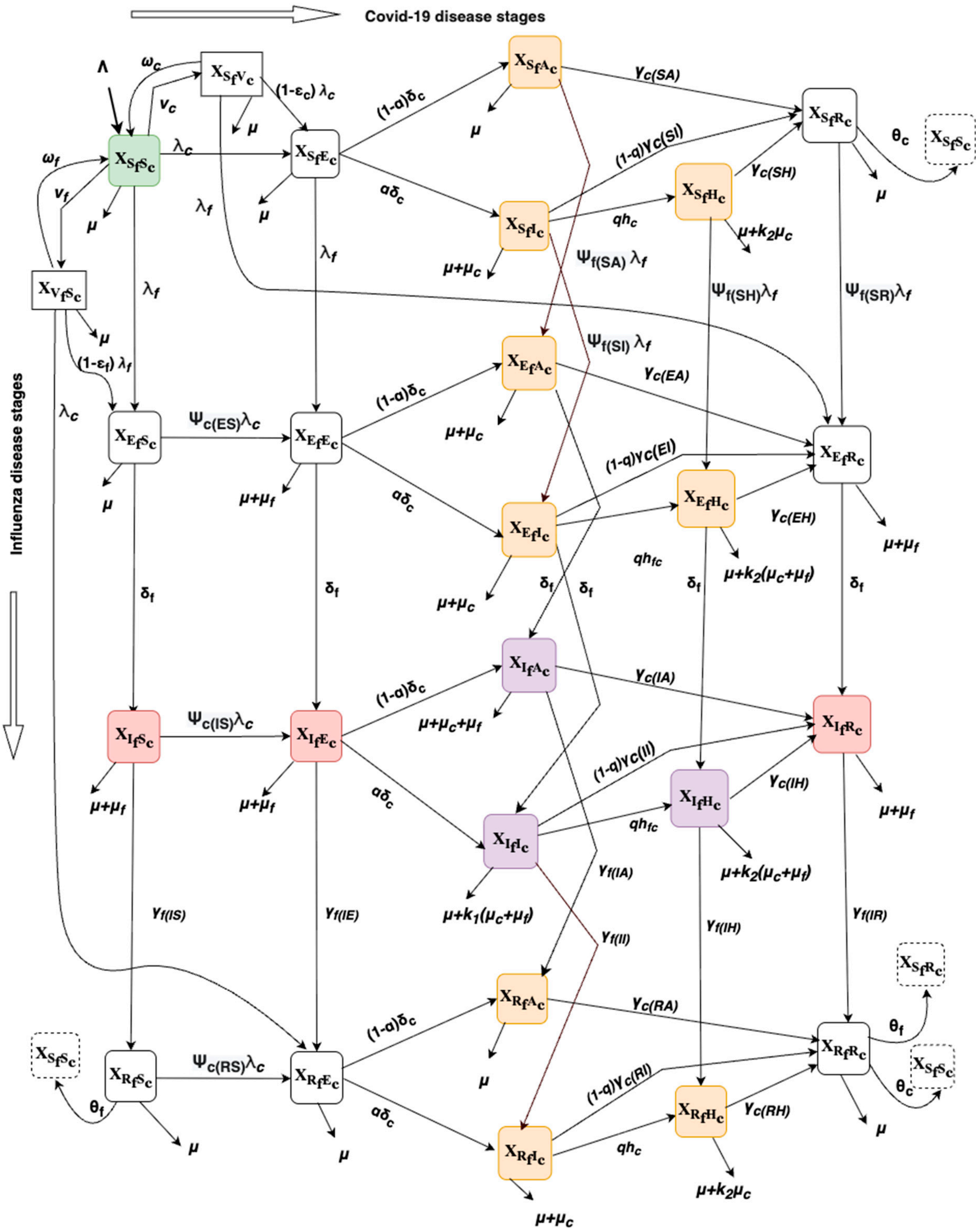

Figure S2: Flow diagram of the *SVEIR-SVEAIHR* co-infection model**.** This model is used as the **sensitivity analysis model (System S2)** to test the findings of the primary SVIR-based model presented in the main text. Its key structural difference is the inclusion of an explicit latent (Exposed) period for influenza (the

$X_{E_f j}$ compartments). Compartments are color-coded by infectious status: influenza-only (red), COVID-19-only (light yellow), and co-infectious (purple).

$$\frac{dX_{S_fS_c}}{dt} = \Lambda + \theta_c(X_{S_fR_c} + X_{R_fR_c}) + \theta_f X_{R_fS_c} + \omega_c X_{S_fV_c} + \omega_f X_{V_fS_c} - \left(\frac{\beta_f I_F}{N} + \frac{\beta_c I_C}{N} + v_c + v_f + \mu\right) X_{S_fS_c}$$

$$\frac{dX_{S_fV_c}}{dt} = v_c X_{S_fS_c} - \left((1-\epsilon_c)\frac{\beta_c}{N} I_C + \frac{\beta_f}{N} I_F + w_c + \mu\right) X_{S_fV_c}$$

$$\frac{dX_{S_fE_c}}{dt} = (1-\epsilon_c)\frac{\beta_c}{N} I_C X_{S_fV_c} + \frac{\beta_c I_C X_{S_fS_c}}{N} - \left(\beta_f \frac{I_F}{N} + \delta_c + \mu\right) X_{S_fE_c}$$

$$\frac{dX_{S_fA_c}}{dt} = (1-\alpha_c)\delta_c X_{S_fE_c} - \left(\psi_{f(SA)}\beta_f \frac{I_F}{N} + \gamma_{c(A)} + \mu\right) X_{S_fA_c}$$

$$\frac{dX_{S_fI_c}}{dt} = \alpha_c \delta_c X_{S_fE_c} - \left(\psi_{f(SI)}\beta_f \frac{I_F}{N} + q h_c + (1-q)\gamma_{c(S)} + \mu + \mu_c\right) X_{S_fI_c}$$

$$\frac{dX_{S_fH_c}}{dt} = q h_c X_{S_fI_c} - \left(\psi_{f(SH)}\beta_f \frac{I_F}{N} + \mu + \kappa_2 \mu_c + \gamma_{c(H)}\right) X_{S_fH_c}$$

$$\frac{dX_{S_fR_c}}{dt} = \gamma_{c(A)} X_{S_fA_c} + \theta_f X_{R_fR_c} + (1-q)\gamma_{c(S)} X_{S_fI_c} + \gamma_{c(H)} X_{S_fH_c} - \left(\psi_{f(SR)}\beta_f \frac{I_F}{N} + \mu + \theta_c\right) X_{S_fR_c}$$

$$\frac{dX_{V_fS_c}}{dt} = v_f X_{S_fS_c} - \left((1-\epsilon_f)\frac{\beta_f}{N} I_F + \frac{\beta_c}{N} I_C + w_f + \mu\right) X_{V_fS_c}$$

$$\frac{dX_{E_fS_c}}{dt} = (1-\epsilon_f)\frac{\beta_f I_F X_{V_fS_c}}{N} + \frac{\beta_f I_F X_{S_fS_c}}{N} - \left(\psi_{c(ES)}\beta_c \frac{I_C}{N} + \delta_f + \mu\right) X_{E_fS_c}$$

$$\frac{dX_{E_fE_c}}{dt} = \psi_{c(ES)}\beta_c \frac{I_C}{N} X_{E_fS_c} + \beta_f \frac{I_F X_{S_fE_c}}{N} - (\delta_c + \delta_f + \mu + \mu_f) X_{E_fE_c}$$

$$\frac{dX_{E_fA_c}}{dt} = (1-\alpha_c)\delta_c X_{E_fE_c} + \psi_{f(SA)}\beta_f \frac{I_F X_{S_fA_c}}{N} - (\zeta_c \gamma_{c(A)} + \delta_f \gamma_f + \mu + \mu_c + \mu_f) X_{E_fA_c}$$

$$\frac{dX_{E_fI_c}}{dt} = \alpha_c \delta_c X_{E_fE_c} + \psi_{f(SI)}\beta_f \frac{I_F X_{S_fI_c}}{N} - \left(q h_{fc} + (1-q)\zeta_c \gamma_{c(S)} + \delta_f + \mu + (\mu_c + \mu_f)\right) X_{E_fI_c}$$

$$\frac{dX_{E_fH_c}}{dt} = q h_{fc} X_{E_fI_c} + \psi_{f(SH)}\beta_f \frac{I_F X_{S_fH_c}}{N} - \left(\delta_f + \mu + \kappa_2(\mu_c + \mu_f) + \zeta_c \gamma_{c(H)}\right) X_{E_fH_c}$$

$$\frac{dX_{E_fR_c}}{dt} = \frac{\beta_f I_F X_{S_fV_c}}{N} + \psi_{f(SR)}\beta_f \frac{I_F X_{S_fR_c}}{N} + \zeta_c \left(\gamma_{c(A)} X_{E_fA_c} + (1-q)\gamma_{c(S)} X_{E_fI_c} + \gamma_{c(H)} X_{E_fH_c}\right) - (\delta_f + \mu + \mu_f) X_{I_fR_c}$$

$$\frac{dX_{I_fS_c}}{dt} = \delta_f X_{E_fS_c} - \left(\psi_{c(IS)}\beta_c \frac{I_C}{N} + \gamma_f + \mu + \mu_f\right) X_{I_fS_c}$$

$$\frac{dX_{I_fE_c}}{dt} = \psi_{c(IS)}\beta_c \frac{I_C X_{I_fS_c}}{N} + \delta_f X_{E_fE_c} - (\delta_c + \gamma_f + \mu + \mu_f) X_{I_fE_c}$$

$$\frac{dX_{I_fA_c}}{dt} = (1-\alpha)\delta_c X_{I_fE_c} + \delta_f X_{E_fA_c} - (\gamma_{c(A)} + \gamma_f) X_{I_fA_c}$$

$$\frac{dX_{I_fI_c}}{dt} = \alpha_c \delta_c X_{I_fE_c} + \delta_f X_{E_fI_c} - \left(q h_{fc} + (1-q)\zeta_c \gamma_{c(S)} + \zeta_f \gamma_f + \mu + \kappa_1(\mu_c + \mu_f)\right) X_{I_fI_c}$$

$$\frac{dX_{I_fH_c}}{dt} = q h_{fc} X_{I_fI_c} + \delta_f X_{E_fH_c} - \left(\gamma_{c(H)} + \zeta_f \gamma_f + \mu + \kappa_2(\mu_c + \mu_f)\right) X_{I_fH_c}$$

$$\frac{dX_{I_fR_c}}{dt} = \gamma_{c(A)} X_{I_fA_c} + (1-q)\zeta_c \gamma_{c(S)} X_{I_fI_c} + \gamma_{c(H)} X_{I_fH_c} + \delta_f X_{E_fH_c} - (\gamma_f + \mu + \mu_f) X_{I_fR_c}$$

$$\frac{dX_{R_fS_c}}{dt} = \gamma_f X_{I_fS_c} - \psi_{c(RS)}\left(\frac{\beta_c I_C}{N} + \mu + \theta_f\right) X_{R_fS_c}$$

$$\frac{dX_{R_fE_c}}{dt} = \beta_c \frac{I_C X_{V_fS_c}}{N} + \psi_{c(RS)}\beta_c \frac{I_C X_{R_fS_c}}{N} + \gamma_f X_{I_fE_c} - (\delta_c + \mu) X_{R_fE_c}$$

$$\frac{dX_{R_fA_c}}{dt} = (1-\alpha_c)\delta_c X_{R_fE_c} + \zeta_f \gamma_f X_{I_fA_c} - (\gamma_{c(A)} + \mu) X_{R_fA_c}$$

$$\frac{dX_{R_fI_c}}{dt} = \alpha_c \delta_c X_{R_fE_c} + \zeta_f \gamma_f X_{I_fI_c} - (q h_c + (1-q)\gamma_{c(S)} + \mu + \mu_c) X_{R_fI_c}$$

$$\frac{dX_{R_fH_c}}{dt} = q h_c X_{R_fI_c} + \zeta_f \gamma_f X_{I_fH_c} - (\mu + \kappa_2 \mu_c + \gamma_{c(H)}) X_{R_fH_c}$$

$$\frac{dX_{R_fR_c}}{dt} = \gamma_{c(A)} X_{R_fA_c} + (1-q)\gamma_{c(S)} X_{R_fI_c} + \gamma_{c(H)} X_{R_fH_c} + \gamma_f X_{I_fR_c} - (\mu + \theta_c + \theta_f) X_{R_fR_c}$$

where, $I_F = X_{I_fS_c} + X_{I_fE_c} + X_{I_fA_c} + X_{I_fI_c} + \eta_{H_c}X_{I_fH_c} + X_{I_fR_c}$ and
$I_C = \eta_{A_c}X_{S_fA_c} + \eta_{I_c}X_{S_fI_c} + \eta_{H_c}X_{S_fH_c} + \eta_{A_c}X_{I_fA_c} + \eta_{I_c}X_{I_fI_c} + \eta_{H_c}X_{I_fH_c} + \eta_{A_c}X_{R_fA_c} + \eta_{I_c}X_{R_fI_c} + \eta_{H_c}X_{R_fH_c}$
respectively are the total number of Influenza infectious and COVID-19 infectious population at a particular instance of time (when infectious modification parameters $\eta_{A_c} = \eta_{I_c} = \eta_{H_c} = 1$).

S1.3. Model Parameterization

The parameters β_c represent the transmission rate of COVID-19, δ_c represents the rate at which patients exposed to COVID-19 become infectious (symptomatic or asymptomatic) with α_c proportion of those exposed to COVID-19 become symptomatic and $(1-\alpha_c)$ become asymptomatic. $\gamma_{c(A)}$, $\gamma_{c(S)}$, and $\gamma_{c(H)}$ respectively represent the recovery rate of the COVID-19 infected from the asymptomatic compartment, the symptomatic compartment, and the hospitalized compartment. The parameter q represents the fraction of COVID-19 infected with symptoms that were admitted to the hospital; h_c, h_{fc} represents the reciprocal of time delays in hospitalization of single infected or co-infected individuals. Likewise, β_f and γ_f respectively represent the transmission rate and the recovery rate of influenza.

In addition, we introduce parameters ψ_f, ψ_c to quantify the varying degree of susceptibility of getting co-infection (or secondary infection) with another pathogen after being infected with (or recovering from) the first pathogens. $\psi_f > 1$ or $\psi_c > 1$ signifies increased susceptibility whereas $\psi_f < 1$ and $\psi_c < 1$ signifies decreased susceptibility). Due to the absence of empirical estimates for these interaction terms—a challenge we identified as a widespread issue in the co-infection modeling literature [5]—we set both to 1 in our baseline to impose neutrality, and vary them independently over [0.7, 1.5] in sensitivity runs (Section 3). Furthermore, co-infected individuals may recover at a slower rate because of compromised or potentially overwhelmed immune systems, so we incorporate these differences into our model by introducing parameters ζ_c and ζ_f. These too are assumed ≥1; baseline $\zeta_c = \zeta_f = 1$. Likewise, recovered people can be re-susceptible to the same pathogen, which is addressed by parameters θ_c, and θ_f. Further, we use v_c, and v_f to represent the daily vaccination rate against COVID-19 and influenza respectively, and use ϵ_c, and ϵ_f to represent the efficacy of those vaccines. Also, the current vaccines available for COVID-19 and influenza are not perfect and do not provide lifelong immunity; therefore, we incorporate these into our model through the parameters ω_c and ω_f accounting for the vaccine-acquired immunity waning.

S1.4. Parameter values and Initial Conditions

The model parameters in our study can be grouped mainly into three types: (1) related to influenza, (2) related to COVID-19, and (3) related to co-infections. The parameters on groups (1) and (2) are readily available in literature or can be extracted from data, but the parameter values related to co-infections of influenza and COVID-19 are not available, or very few or are not appropriate for our model construction. Therefore, in this study, we considered the influenza and COVID-19 parameter values from the published literature and assumed the co-infection parameter based on our insight. Likewise, for the initial condition of the state variables, we initialize all living compartments as fractions of the population (so the living mass sums to 1). At $t = 0$ we set $X_{S_fS_c} = 0.99099$, $X_{S_fE_c} = 0.003$, $X_{S_fA_c} = X_{S_fI_c} = 0.001$, $X_{S_fH_c} = X_{S_fR_c} = 0.0$, $X_{I_fS_c} = 0.004$; the co-infection seeds $X_{I_fE_c} = X_{I_fA_c} = X_{I_fI_c} = X_{I_fH_c} = X_{I_fR_c} = 10^{-6}$; and the remaining recovered classes $X_{R_fS_c} = X_{R_fE_c} = X_{R_fA_c} = X_{R_fI_c} = X_{R_fH_c} = X_{R_fR_c} = 0.0$. Thus, at baseline ~0.3% are COVID-exposed, 0.1% are COVID-asymptomatic, 0.1% are COVID-symptomatic, and 0.4% are influenza-infectious; no one starts vaccinated or recovered. Co-infected states are initialized at 10^{-6} to allow invasion without imposing a finite concurrent prevalence.

The parameters, their definitions, baseline values, units, and their source are displayed in Table (S1). Likewise, the initial condition of state variables is included in Table (S2). Note that we consider a range for transmission values to reflect temporal variability; unless stated otherwise the baselines are $\beta_f = 0.4$ and $\beta_c = 0.4725$, which calibrate $\mathcal{R}_0 \approx 2.0$ for both pathogens, enabling like-for-like comparisons of dynamics.

Table S1: Model Parameters, their definitions, and their values

Parameter	Definition	Value(unit)	Source(s)
μ	Natural mortality rate	$\frac{1}{(80 \times 365)} (day)^{-1}$	Assumed
β_c	Transmission rate of COVID-19	$[0,1]\ (day)^{-1}$	Varied
δ_c	Rate of getting infectious once exposed to COVID-19	$\frac{1}{5} (day)^{-1}$	[6,7]
δ_f	Rate of getting infectious once exposed to influenza (Used in the *SVEIR* sensitivity model, System S2)	$\frac{1}{1.5} (day)^{-1}$	[8]
α_c	Proportion of COVID-19 exposed developing symptoms	0.7	[9]
q	Proportion of symptomatic infected going to hospital	0.0624	[10]
h_c	Hospitalization rate of COVID-19 symptomatic	$\frac{1}{4} (day)^{-1}$	Assumed
$\kappa_1(\kappa_2)$	Factors affecting deaths of symptomatic co-infected(hospitalized) patients compared to that of noncritical cases	3(6)	[11]
$\gamma_{c(A)}$	Recovery rate of COVID-19 asymptomatic infected	$\frac{1}{7} (day)^{-1}$	[12]
$\gamma_{c(I)}$	Recovery rate of symptomatic COVID-19 infected	$\frac{1}{10} (day)^{-1}$	[11]
$\gamma_{c(H)}$	Recovery rate of hospitalized COVID-19 infected	$\frac{1}{9} (day)^{-1}$	[9]
μ_c	Disease induced mortality rate (COVID-19)	$0.001\ (day)^{-1}$	[11]
ν_c	Vaccination rate for COVID-19	$0.005\ (day)^{-1}$	Assumed
ϵ_c	Efficacy of COVID-19 vaccine	0.95	[11,13]
ω_c	Waning rate COVID-19 vaccine	$\frac{1}{180} (day)^{-1}$	[11]
θ_c	Immunity waning rate of COVID-19 recovered patients	$\frac{1}{365}$ {(day}$^{-1}$)	[11]
η_{A_c}	Infectious adjustment parameter (asymptomatic infected compared to symptomatic infected)	0.7228	[14]
η_{I_c}	Infectious adjustment parameter (symptomatic infected accounting their assumed isolation)	0.35	Assumed
η_{H_c}	Infectious adjustment parameter (hospitalized infected compared to symptomatic infected)	0.6526	[14]
β_f	Transmission rate of influenza	$[0,1]\ (day)^{-1}$	Varied

γ_f	Recovery rate of influenza	$\frac{1}{5}\,(day)^{-1}$	[10]
ψ_f, ψ_c	Susceptibility modifier for secondary infection post-recovery/infection from the primary pathogen	Varied	Assumed
ζ_f, ζ_c	change in recovery time of co-infected	≥ 1	Assumed
v_f	Vaccination rate(influenza)	$0.005\ (day)^{-1}$	Assumed
ϵ_f	Efficacy of influenza vaccine	0.632	[9]
ω_f	Vaccination waning rate(influenza)	$\frac{1}{180}\,(day)^{-1}$	[11]
θ_f	Immunity waning rate of influenza recovered	$\frac{1}{365}\,(day)^{-1}$	[11]
μ_f	Disease induced mortality rate (influenza)	$0.001\ (day)^{-1}$	[9]

Table S2: Model State variables and their initial seeded values

State variables	Description	Value
$X_{S_fS_c}$	Susceptible to both Flu and COVID-19	0.990995
$X_{V_fS_c}, X_{S_fV_c}$	Vaccinated for one virus, susceptible to the other	0.0 (each)
$X_{S_f\mathrm{E}_c}$	Susceptible to Flu, Exposed to COVID-19	0.003
$X_{S_fH_c}, X_{S_fR_c}$	Susceptible to Flu, Hospitalized (Recovered) to COVID-19	0.0 (each)
$X_{S_fA_c}, X_{S_fI_c}$	Susceptible to Flu, Asymptomatic/Symptomatic with COVID-19	0.001 (each)
$X_{I_fE_c}, X_{I_fA_c}, X_{I_fI_c}, X_{I_fH_c}, X_{I_fR_c}$	Co-infected states (e.g., Flu-Infectious, COVID-Exposed)	10^{-6} (each)
$X_{I_fS_c}$	Infectious with Flu, Susceptible to COVID-19	0.004
$X_{R_fS_c}$	Flu recovered, Susceptible to COVID-19	0.0
$X_{R_fE_c}, X_{R_fA_c}, X_{R_fI_c}, X_{R_fH_c}$	Flu recovered, Infected to COVID-19	0.0 (each)
$X_{R_fR_c}$	Recovered to both	0.0
N	Total population	1.0

S2. Mathematical Formulation of Incidence decomposition and Burden Accounting

The *total daily incidence* is the sum of the target-side infection terms in the ODE. We then partition this incidence by *pathway* and by *source*.

S2.1 Pathway-specific incidence (per pathogen).

We partition each pathogen's total per-day incidence into epidemiologically meaningful transmission pathways. Sequential (strict) denotes infections that occur after recovery from the other virus—for COVID-19, the flow from individuals recovered from influenza to COVID-19 exposed $\left(X_{R_f S_c} \to X_{R_f E_c}\right)$; for influenza, the flow from individuals recovered from COVID-19 to influenza infected $\left(X_{S_f R_c} \to X_{I_f R_c}\right)$. Overlapping (concurrent) aggregates infections acquired while currently infected with the other virus—for COVID-19, the flow from influenza-infected to co-infected $(X_{I_f S_c} \to X_{I_f E_c})$; for influenza, the flows from any COVID-19 disease stage to a co-infected stage (e.g., $X_{S_f E_c} \to X_{I_f E_c}, X_{S_f A_c} \to X_{I_f A_c}$). We compute pathway-specific rates directly from system (S1), and integrate over 180 days with the composite trapezoid rule (1-day grid) with units are fractions of the population.

S2.2 Source-decomposed incidence.

We split the force of infection into additive source classes and weight each by the same target-side susceptibility mass that appears in system (S1) (including vaccine and cross-susceptibility modifiers).

Let $X_{ij}(t)$ denote the population in compartment with influenza status $i \in S_f, V_f, I_f, R_f$ and COVID-19 status $j \in S_c, V_c, E_c, A_c, I_c, H_c, R_c$. For pathogen $p \in c, f$ and source $s \in$ single,co,post- ($\ominus$ means "the other virus"), define the source-specific force of infection

$$\lambda_p^{(s)}(t) \;=\; \beta_p \sum_{(i,j)\in \mathcal{J}_p^{(s)}} \eta_{ij}\, X_{ij}(t),$$

where β_p is the transmission rate for pathogen p, and η_{ij} is the infectiousness multiplier for compartment (i,j) (e.g., distinct scalings for A_c, I_c, H_c on the COVID side). The total force of infection decomposes additively,

$$\lambda_p(t) \;=\; \lambda_p^{(\text{single})}(t) + \lambda_p^{(\text{co})}(t) + \lambda_p^{(\text{post-}\ominus)}(t).$$

We attribute *incidence* to sources by weighting each $\lambda_p^{(s)}$ with the same target-side susceptibility mass that appears in system (S1):

$$\iota_p^{(s)}(t) \;=\; \lambda_p^{(s)}(t)\; \mathcal{M}_{\mathscr{p}}(t), \qquad \mathcal{M}_p(t) \;\equiv\; \sum_{q\in \mathcal{T}_p} w_q\; X_q(t).$$

Here $\mathcal{T}_{\mathscr{p}}$ is the set of target-side compartments that receive new infections with pathogen p in (S1), and w_q are their per-target susceptibility weights (e.g., 1 for fully susceptible, $1-\varepsilon_p$ for vaccinated, $\psi_{\ominus\to p}$ for recovered-from-other, etc.). Cumulative, source-attributed incidence over $[0,T]$ is

$$I_p^{(s)} \;=\; \int_0^T \iota_p^{(s)}(t)\; dt, \qquad I_p \;=\; \sum_s I_p^{(s)}.$$

Explicit infectious-source sets. Using our X_{ij} notation, the infectious "source" collections are:

COVID-19 sources ($p = c$, infectious statuses $j \in A_c, I_c, H_c$):

$$\mathcal{J}_c^{(\text{single})} = (i,j): i \in S_f, V_f,\ j \in A_c, I_c, H_c,$$
$$\mathcal{J}_c^{(\text{co})} = \left(I_f, j\right): j \in A_c, I_c, H_c,$$
$$\mathcal{J}_c^{(\text{post-}f)} = \left(R_f, j\right): j \in A_c, I_c, H_c.$$

for COVID–19,

$$\lambda_c(t) = \lambda_c^{single}(t) + \lambda_c^{co}(t) + \lambda_c^{post\text{-}flu}(t),$$

where "single" collects infectious $X_{S_f A_c}, X_{S_f I_c}, X_{S_f H_c}$, "co" collects $X_{I_f A_c}, X_{I_f I_c}, X_{I_f H_c}$, and "post-flu" collects $X_{R_f A_c}, X_{R_f I_c}, X_{R_f H_c}$ (with the appropriate η scalings). An analogous decomposition is used for influenza.

Influenza sources ($p = f$, infectious status $i = I_f$):

$$\mathcal{J}_f^{(\text{single})} = \left(I_f, j\right): j \in S_c, V_c,$$
$$\mathcal{J}_f^{(\text{co})} = \left(I_f, j\right): j \in E_c, A_c, I_c, H_c,$$
$$\mathcal{J}_f^{(\text{post-}c)} = \left(I_f, R_c\right).$$

Notes: (i) By construction, $\iota_p(t) = \sum_s \iota_p^{(s)}(t)$. (ii) The same vaccine and cross-susceptibility modifiers used in (S1) are applied inside $\mathcal{M}_p(t)$, so the accounting is exactly consistent with the ODE infection terms.

S2.3 Disease burden

We define *disease burden* as the total person–days of illness over the analysis horizon $[0, T]$ obtained by integrating the mass in all *actively ill* compartments for each pathogen. With population fractions ($N = 1$), the units are fraction–days. All illness states are equally weighted (unweighted burden).

COVID-19 burden: Let the COVID-illness compartments be

$$\mathcal{J}_c = \underbrace{\{X_{S_f A_c}, X_{S_f I_c}, X_{S_f H_c}\}}_{\text{singly infected (COVID only)}} \cup \underbrace{\{X_{I_f A_c}, X_{I_f I_c}, X_{I_f H_c}\}}_{\text{co-infected}} \cup \underbrace{\{X_{R_f A_c}, X_{R_f I_c}, X_{R_f H_c}\}}_{\text{recovered-from-flu} \to \text{COVID}}.$$

The COVID burden is $B_c = \int_0^T \sum_{X \in \mathcal{J}_c} X(t)\, dt.$

Influenza burden: Let the influenza-illness compartments be

$$\mathcal{J}_f = \underbrace{X_{I_f S_c}}_{\text{singly infected (influenza only)}} \cup \underbrace{\{X_{I_f E_c}, X_{I_f A_c}, X_{I_f I_c}, X_{I_f H_c}\}}_{\text{co-infected}} \cup \underbrace{X_{I_f R_c}}_{\text{recovered-from-COVID} \to \text{influenza}}.$$

The influenza burden is $B_f = \int_0^T \sum_{X \in \mathcal{J}_f} X(t)\, dt.$

S2.4. Interventions

Interventions act multiplicatively on infection terms. Non-pharmaceutical interventions (NPIs) implement a uniform contact reduction $\beta_j \mapsto (1 - u_1)\beta_j, j \in \{c, f\}$. Vaccination modifies the susceptibility of vaccinated targets via $\left(1 - u_4\, \varepsilon_j\right)$, with coverage (flow into the vaccinated class) v_j and waning w_j. These same modifiers are applied both in the ODE system (S1) and in all incidence/accounting formulas in Section 2.3.

S2.5. Numerical Integration and units

Cumulative quantities are obtained by integrating the relevant per-day rates with the composite trapezoid rule on a 1-day grid over the analysis horizon. Units are reported as *fractions of the population* (with $N = 1$). Also, we note that for clarity and focus, all the primary analysis presented in the results section will use the baseline scenario where both diseases are equally transmissible ($\mathcal{R}_{0c} = \mathcal{R}_{0f} = 2.0$))

S3. Sensitivity to Model Framework and Transmissibility (The Methods)

To assess the robustness of our central findings regarding the asymmetric transmission dynamics, we performed two specific sensitivity analyses as described in the main text's Methods section.

S3.1 Model Structure Sensitivity (*SVIR* vs. *SVEIR* for Influenza)

We compared the outcomes from our primary *SVIR*-based model (System S1, described in Section S1.1). The *SVEIR* variant (System S2) was derived from System S1 by:

i) Adding influenza Exposed compartments (i.e., $X_{E_f j}$ for all j).

ii) Rerouting all new influenza infections directly into these corresponding Exposed compartments.

iii) Adding the progression rate parameter δ_f (the latent period for flu).

All other parameters, including the influenza transmission rate β_f (calibrated to achieve the target R_{0f} for the respective model structure) and recovery rate γ_f, as well as all COVID-19 parameters and initial conditions, were kept identical between the two model simulations. We simulated both the *SVEIR-SVEAIHR* and *SVIR-SVEAIHR* models under the baseline parameter set ($R_{0c} = 2.0, R_{0f} = 2.0$).

S3.2 Relative Transmissibility Sensitivity (R_0 Pair Analysis)

We simulated the primary *SVIR-SVEAIHR* model (System S1) under three distinct scenarios defined by target pairs of basic reproduction numbers (R_{0c}, R_{0f}):

i) Higher Influenza Transmissibility: ($R_{0c} = 1.5, R_{0f} = 2.0$)

ii) Equal Baseline Transmissibility: ($R_{0c} = 2.0, R_{0f} = 2.0$)

iii) Higher COVID-19 Transmissibility: ($R_{0c} = 2.0, R_{0f} = 1.5$)

These target R_0 values were achieved by adjusting only the pathogen-specific transmission rate parameters, β_c and β_f, respectively. All other model parameters (durations of latent/infectious periods, recovery rates, vaccine parameters if used, interaction modifiers ψ, etc.) and initial conditions were held constant at their baseline values as listed in Supplementary Table S1 and S2. For each of the three (R_{0c}, R_{0f}) scenarios, the model was simulated for 180 days. The primary outcomes collected were the cumulative source contributions (Singly-infected, Co-infected, Sequentially-infected) and pathway contributions (Primary/Direct, Sequential, Overlapping) to the total incidence of both COVID-19 and influenza, calculated using the decomposition framework detailed in Section S2. These decomposition results were then compared across the three scenarios to evaluate the robustness and sensitivity of the asymmetric findings to relative transmissibility. The specific β_c, β_f values used and the resulting decomposition percentages are presented in the main text's Results section and Supplementary Section S4.

S3.3 Global Sensitivity Analysis

To understand the parameter drivers of the model (System S1), we quantified the global sensitivity of model outcomes to uncertain parameters using Latin hypercube sampling (LHS) and partial rank correlation coefficients (PRCCs). Parameters were sampled independently from broad uniform ranges centered on their baselines (Table S1). For each virus, we simulated n = 500 parameter sets over 180 days and recorded two primary outcomes: (i) cumulative incidence and (ii) total person-days of illness ("burden"); pathway shares were retained as secondary descriptors. To summarize influence, we computed PRCCs between each input parameter and each outcome after rank-transforming variables and adjusting for the remaining parameters. Ninety-five percent confidence intervals for PRCCs were obtained by percentile bootstrapping with B = 600 resamples; effects with intervals excluding zero were interpreted as materially associated with the outcome.

Because PRCC assumes monotone associations, we verified this a posteriori for the leading factors by plotting rank-partial residuals and fitting isotonic curves; fits were strongly monotone for top drivers, while a few weak effects showed wide intervals and occasional non-monotonicity flags. For interpretability, PRCC sign indicates direction (protective vs amplifying) and magnitude provides a relative ranking rather than an absolute effect size; tornado plots present the full ordering. As a specificity check, we repeated the analysis under a decoupled control in which sequence modification was disabled and co-infection/post-other-virus contributions were removed from the accounting; this distinguishes genuine cross-pathway mediation from spurious correlations.

In this model, monotonicity held for a subset of parameters (e.g., for COVID incidence: $\beta_c, \gamma_{cA}, \gamma_{cI}, \eta_A, \eta_I, \psi_c$) and failed for others (e.g., $\beta_f, \gamma_f, \psi_f$, hospitalization/triage modifiers).

Robustness checks We repeated LHS with log-scaling for strictly positive rates, increased the sample size (n ≥1000), and confirmed that the qualitative rankings were robust.

S4. Key Findings from Sensitivity Analysis (The Results)

S4.1 Findings from Model Structure Sensitivity

As shown in Supplement Figure S7, the structure of the influenza model had a notable impact on the epidemic dynamics. Removing the latent period for influenza (the primary SVIR model) resulted in an earlier and sharper influenza peak compared to the SVEIR structure. This shift in influenza timing directly impacted the subsequent COVID-19 wave by altering the timing and size of the influenza-recovered pool available for sequential infection.

Consequently, the quantitative contribution of the sequential pathway to COVID-19 incidence was modulated (a finding summarized in the main text's Table 1). However, across both model structures simulated at the baseline ($R_{0c} = 2.0, R_{0f} = 2.0$), the primary qualitative finding remained consistent: COVID-19 transmission was dominated by the sequential source/pathway, while influenza transmission was dominated by the singly-infected source/pathway. This confirms that the observed asymmetry is a core feature of the co-epidemic dynamics and not an artifact of including or not the influenza latent period.

S4.2 Findings from Relative Transmissibility Sensitivity

The analysis of the R_0 pairs for our primary *SVIR* model reveals a consistent and predictable pattern in the co-epidemic dynamics. In all scenarios, influenza transmission remains overwhelmingly driven by singly-

infected individuals, while the transmission driver for COVID-19 is highly sensitive to the relative transmissibility of the two viruses. The results (detailed in the main text's Table 1) are:

***Baseline (Equal Transmissibility**: $\boldsymbol{R_{0c} = 2.0, R_{0f} = 2.0}$*):

- **COVID-19**: The sequential pathway (from influenza-recovered individuals) accounts for 73.4% of transmission and 76.1% of the disease burden.
- **Influenza**: The singly-infected pathway accounts for 96.0% of transmission and 93.4% of the burden. This confirms the strong asymmetry in the baseline case.

Higher Influenza Transmissibility (**$\boldsymbol{R_{0c} = 1.5, R_{0f} = 2.0}$):***

- **COVID-19**: The sequential pathway's contribution is at its highest, increasing to 75.2% of transmission and 76.6% of the burden.
- **Influenza**: The singly-infected pathway becomes even more dominant, accounting for 97.9% of transmission. This scenario demonstrates that a more aggressive influenza wave creates a larger pool of susceptible individuals, maximally amplifying the sequential effect for COVID-19.

Higher COVID-19 Transmissibility (**$\boldsymbol{R_{0c} = 2.0, R_{0f} = 1.5}$):***

- **COVID-19**: The sequential pathway's contribution is at its lowest among the three scenarios considered, dropping to 46.2% of transmission and 50.9% of the burden.
- **Influenza**: The singly-infected pathway's contribution is also at its lowest, though it still accounts for a large majority at 87.6% of transmission. This shows that when COVID-19 is inherently more transmissible, it relies less on the post-influenza population to spread.

The transmission contribution and disease burdens corresponding to different sources and for different pairs of (R_{0c}, R_{0f}) as described above using the model *SVIR–SVEAIHR* are displayed as a histogram below:

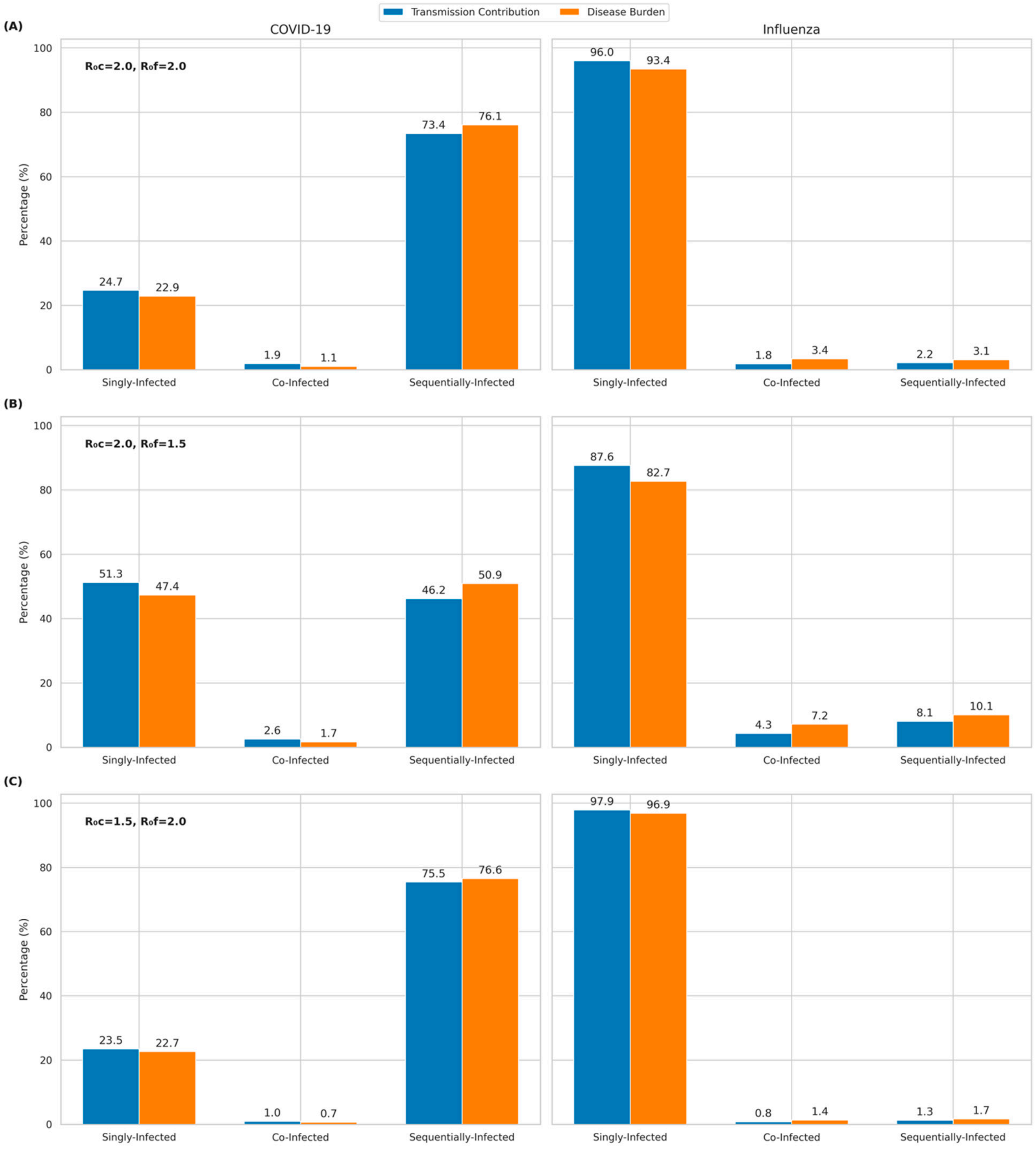

Figure S3 Bar diagram showing the Transmission contribution of different sources and the Burden contribution using models SVIR–SVEAIHR under different pairs of basic reproduction numbers ($\boldsymbol{R_0}$). **A–** ($\boldsymbol{R_{0c} = 2.0, R_{0f} = 2.0}$), **B–** ($\boldsymbol{R_{0c} = 2.0, R_{0f} = 1.5}$) and **C–** ($\boldsymbol{R_{0c} = 1.5, R_{0f} = 2.0}$). First column figures correspond to COVID-19, and the second column corresponds to influenza.

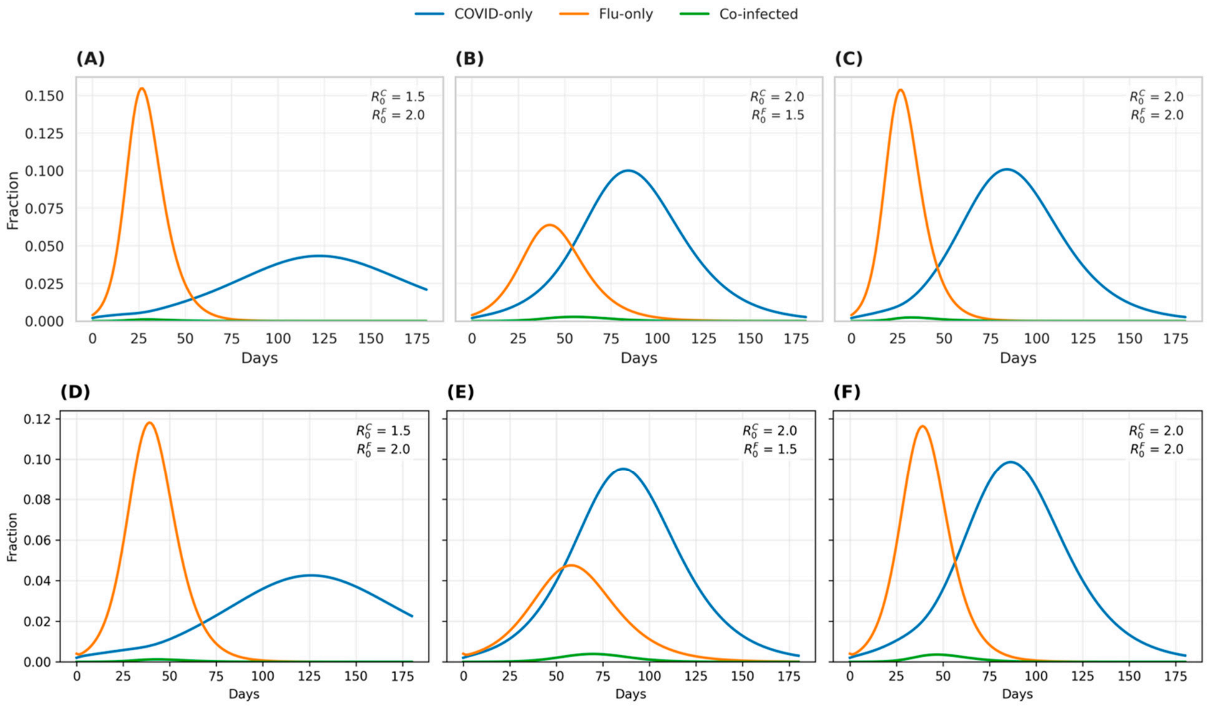

Figure S4 Comparison of epidemic dynamics using *SVIR* vs. *SVEIR* structures for influenza under different pairs of basic reproduction numbers ($\boldsymbol{R_0}$). Curves show prevalence over time for Flu-only, COVID-only, and Co-infected populations for both model variants. *SVIR-SVEAIHR* **(A-C)** and *SVEIR-SVEAIHR* **(D-F)**.

These results provide strong evidence for our paper's central mechanism. The data clearly shows that the size of the sequential COVID-19 epidemic is directly and positively correlated with the size and strength of the preceding influenza epidemic. A more transmissible influenza virus creates a larger reservoir of recovered individuals more quickly, which in turn provides more "fuel" for the sequential COVID-19 pathway.

S4.3 Finding from Global Sensitivity Analysis

COVID-19 outcome (Figure S5(a), Table S3): The dominant positive driver is the transmission rate β_c (PRCC = 0.889 [0.871, 0.907]). Non-pharmaceutical intensity u_1 is strongly protective (PRCC = −0.761 [-0.800, -0.722]), as are the COVID vaccination rollout rate v_c (PRCC = −0.715 [-0.758, -0.667]) and vaccine efficacy ε_c (PRCC = −0.392 [-0.462, -0.315]). Cross-susceptibility from prior influenza, ψ_c, is a substantial amplifier (PRCC = 0.713 [0.664, 0.759]), indicating that increases in sequential risk materially raise the total COVID burden. Faster recovery reduces incidence: $\gamma_{c(A)}$ (PRCC = −0.610 [-0.661, -0.555]) and $\gamma_{c(S)}$ (PRCC = −0.482 [-0.553, -0.412]). Infectiousness scalings η_I (symptomatic) and η_A (asymptomatic) are positively associated (PRCC = 0.672 and 0.604), highlighting calibration importance. The influenza transmission rate β_f exerts a smaller but non-negligible positive cross-effect on COVID (PRCC = 0.279 [0.191, 0.371]) via overlapping/co-infection pathways.

Influenza outcome (Figure S5(b), Table S3): Influenza incidence is dominated by its own transmission and recovery parameters: β_f (PRCC = 0.974 [0.970, 0.978]) and γ_f (PRCC = −0.943 [-0.952, -0.933]). NPIs again show a strong protective association (PRCC for u_1 = −0.883 [-0.906, -0.858]). Influenza vaccination rollout and efficacy are influential—v_f (PRCC = −0.609 [-0.664, -0.551]) and ε_f (PRCC = −0.546 [-0.602, -0.483])—with cross-susceptibility ψ_f contributing a smaller positive effect (PRCC = 0.226 [0.126, 0.317]).

Several cross-coupled COVID parameters (e.g., h_c, δ_c, η_I, η_H) have weak effects with CIs that touch zero, consistent with minor influence on influenza totals under the sampled ranges.

Across both pathogens, the rank ordering is consistent with control levers that primarily act on transmission (NPIs, β) and removal (recovery, vaccination). For COVID-19, cross-infection susceptibility ψ_c ranks comparably to vaccination rate v_c, underscoring that reducing sequential risk (e.g., via timely influenza vaccination) can materially curb COVID transmission in co-circulation. For influenza, the outcome is less sensitive to COVID-side parameters, reflecting a more self-contained epidemic dynamic in the current parameterization. Moreover, as co-infected individuals transmit COVID and prior influenza modifies COVID susceptibility, influenza transmissibility β_f, it exerts a secondary but measurable influence on COVID outcomes (PRCC ≈ 0.28, 95% CI [0.19, 0.37]). When we disable coupling ($\psi_c = \psi_f = 1.0$) and removing co-infected/post-flu contributions from I_C, the PRCC of β_f for COVID collapses toward zero, confirming that this effect is mediated by the concurrent and sequential pathways. These results support prioritizing: (i) measures that depress β_c, β_f (NPIs, behavior change), (ii) accelerated roll-out and high efficacy of the pathogen-matched vaccines ($v_c, v_f, \varepsilon_c, \varepsilon_f$), and (iii) surveillance and mitigation of cross-susceptibility effects (ψ_c, ψ_f).

Table S3: Global sensitivity of cumulative incidence (180 days) to model parameters: LHS–PRCC with 95% CIs (COVID-19 vs. Influenza).

**Param.**	**PRCC (COVID cumulative incidence)**	**95% CI**	**Param.**	**PRCC (Influenza cumulative incidence)**	**95% CI**
β_c	0.889	[0.871, 0.907]	β_f	0.974	[0.970, 0.978]
u_1	-0.761	[-0.800, -0.722]	γ_f	-0.943	[-0.952, -0.933]
v_c	-0.715	[-0.758, -0.667]	u_1	-0.883	[-0.906, -0.858]
ψ_c	0.713	[0.664, 0.759]	v_f	-0.609	[-0.664, -0.551]
η_I	0.672	[0.622, 0.719]	ε_f	-0.546	[-0.602, -0.483]
$\gamma_{c(A)}$	-0.610	[-0.661, -0.555]	ψ_f	0.226	[0.126, 0.317]
η_A	0.604	[0.541, 0.659]	h_c	-0.088	[-0.171, -0.013]
$\gamma_{c(S)}$	-0.482	[-0.553, -0.412]	δ_c	0.081	[-0.008, 0.166]

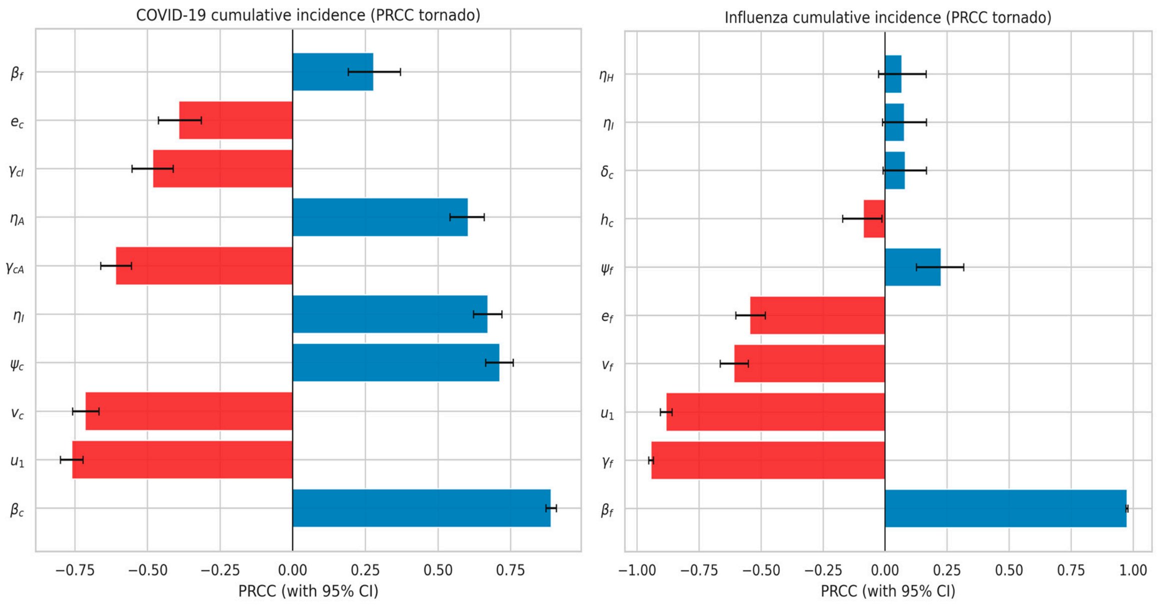

(a) (b)

Figure S5: Cumulative incidence: PRCC tornado with 95% bootstrap CIs. Bars to the right (blue) increase incidence; to the left (red) decrease it. (a) COVID-19 and (b) influenza.

Because several parameters–outcome relationships were non-monotone after adjustment—a common feature in interacting multi-pathogen systems—we interpreted PRCC signs and magnitudes only where monotonicity held and used distance correlation/HSIC and partial-dependence curves elsewhere; this mixed approach yields stable qualitative rankings without over-interpreting PRCC where its assumptions are violated.

S5. Additional Numerical Results

S5.1 Transmission vs. Disease Burdens by Clinical Presentation (COVID-19)

To add a layer of clinical detail to this analysis, our stratified model also allows us to dissect the contributions of different clinical presentations—asymptomatic, symptomatic, and hospitalized—to both the spread of COVID-19 and its overall disease burden. This analysis reveals a critical distinction in the role each group plays in the co-epidemic, as illustrated in **Figure S6**.

Our results indicate that COVID-19 transmission is primarily driven by asymptomatic individuals, who are responsible for 48.9% of all new infections. Symptomatic persons contribute a substantial, but smaller, 44.6% to onward transmission. When analyzing the disease burden (total person-days of illness), the roles are reversed. Symptomatic individuals account for the majority of the total burden at 60.6%, while asymptomatic individuals account for 31.3%. The contribution from hospitalized patients is 6.4% of transmission and 8.1% of the burden, highlighting that while they are not the main drivers of spread, they represent a disproportionately large share of the disease's severity. This finding underscores the dual challenge faced by public health systems: managing the widespread transmission from symptomatic individuals in the community while also handling the severe, resource-intensive burden of those who are hospitalized.

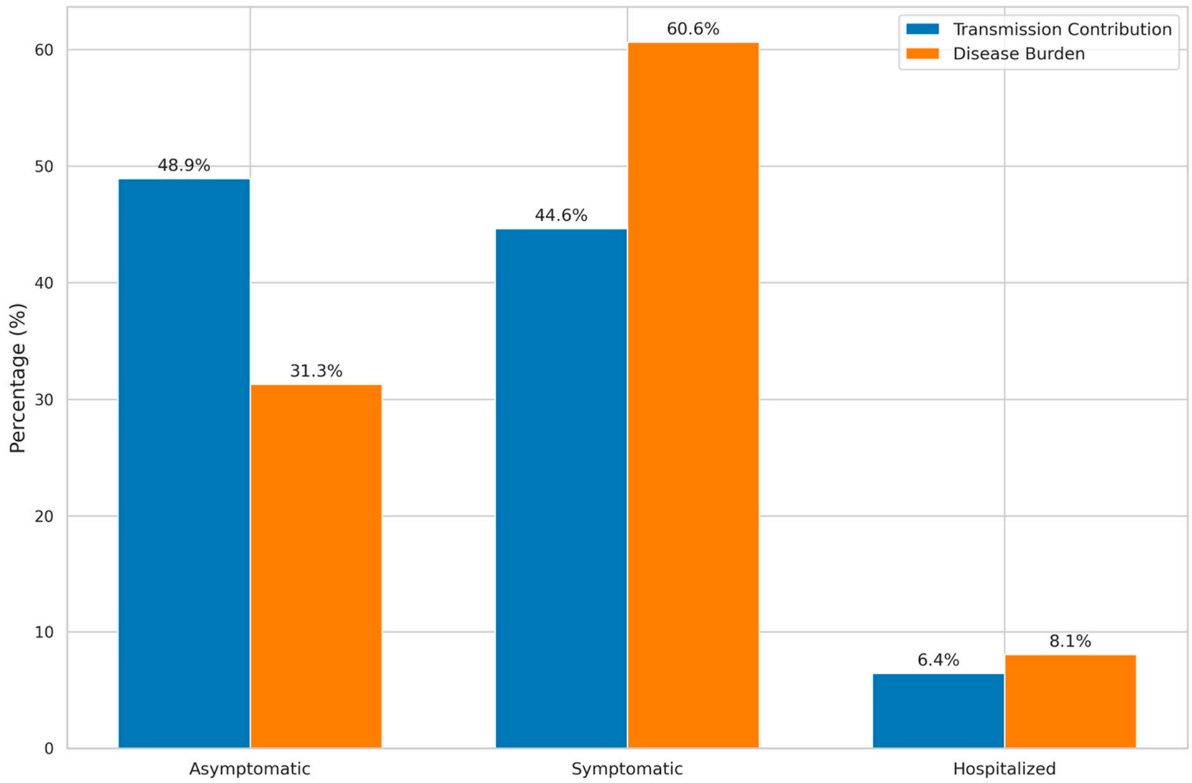

Figure S6 Comparison of contribution to total COVID-19 transmission versus disease burden, broken down by clinical presentation (Asymptomatic, Symptomatic, and Hospitalized). Bars are normalized to 100%. Integrated over 180 days by the composite trapezoid rule.

S5.2 Impact of Epidemic Timing Scenarios

Figure S7 illustrates how the shares of cumulative incidence from each transmission pathway (direct, sequential, overlapping) are affected by the relative introduction timing of the two viruses, with delays of 0, 30, and 60 days.

- **Panel A (Flu-first):** This panel shows the impact on COVID-19 pathways. The sequential pathway (orange bar) is the dominant driver of COVID-19 transmission, regardless of the delay. It accounts for 72.9% of cumulative incidence at a 0-day delay and increases slightly to 78.3% at 30 days and 78.6% at 60 days. The direct pathway (blue bar) and overlapping pathway (green bar) are minor contributors.
- **Panel B (COVID-first):** This panel shows the impact on Influenza pathways. The pathway mix for influenza is highly sensitive to the delay. At a 0-day delay, the epidemic is almost entirely driven by the direct pathway (95.2%). As the delay increases, the sequential share (orange bar) rises markedly from 2.2% (0-day delay) to 12.4% (30-day delay) and 38.3% (60-day delay). The overlapping share (green bar) also increases from 2.6% to 14.6% as the delay extends to 60 days.

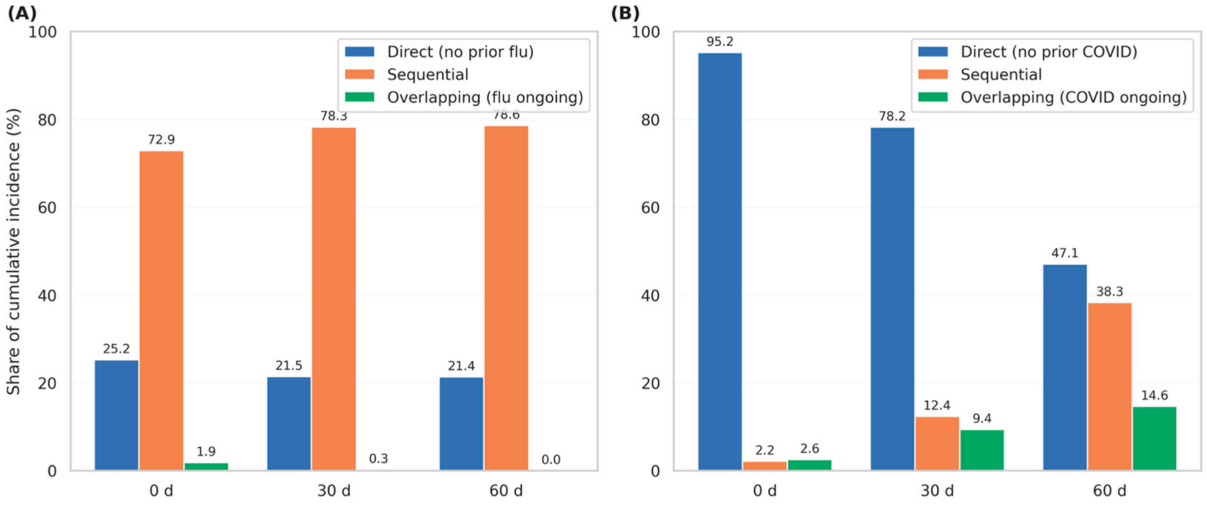

Figure S7 Pathway shares vs. inter-epidemic delay. Share of cumulative incidence by pathway (direct, sequential, overlapping) when one epidemic precedes the other by 0, 30, or 60 days. A: flu-first, showing pathways for COVID-19. B: covid-first, showing pathways for influenza.

S5.3 Effect of Combined Intervention Strategies

Figure S8 illustrates the impact of layering NPIs together with disease specific vaccinations. We simulated five scenarios to compare the cumulative incidence (fraction of population infected over 180 days) for both COVID-19 and influenza.

Relative to the baseline (no interventions), which resulted in a cumulative incidence of 0.799 for COVID-19 and 0.796 for influenza, applying NPIs alone (at 20% reduction) produced moderate declines (COVID-19: 0.618; influenza: 0.640).

Adding a COVID-19 vaccine on top of NPIs sharply reduced COVID-19 incidence to 0.311 with little change in influenza (0.640). Conversely, adding an influenza vaccine to NPIs primarily reduced influenza incidence (to 0.514) with no change in COVID-19 (0.618). Deploying both vaccines alongside NPIs resulted in the smallest combined footprint (COVID-19: 0.335; influenza: 0.527), reinforcing that the combined strategy yields the smallest joint burden.

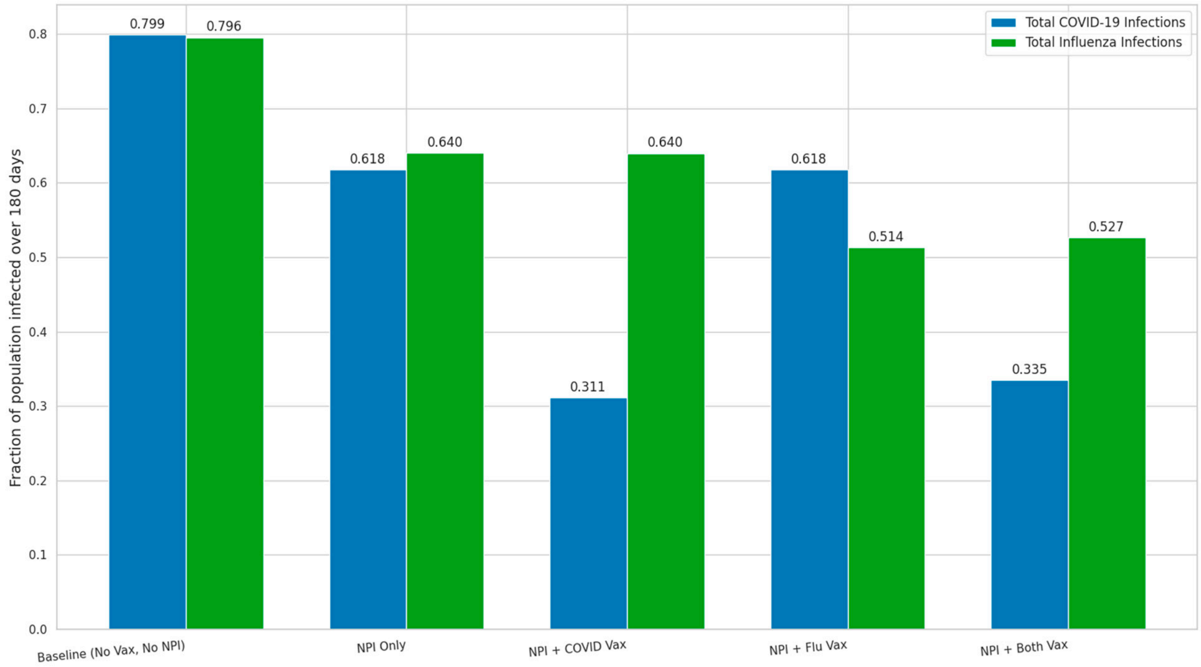

Figure S8: Combined strategies**.** Fractions of the population infected over 180 days under five strategies: baseline, NPI only (20% reduction in effective contacts via e.g. social distancing or masking), NPI+COVID-19 vaccination, NPI+influenza vaccination, and NPI+both vaccines.

S5.4 Sensitivity to Cross-Susceptibility

Figure S9 explores how outcomes change as the cross-susceptibility parameters ψ_c (susceptibility to COVID after flu) and ψ_f (susceptibility to flu after COVID). The parameters were varied independently from 0.6 protective effect) to 1.6 (enhanced susceptibility).

The heatmaps show that the COVID-19 sequential pathway (bottom-left panel) is highly sensitive to ψ_c. As ψ_c increases, the share of COVID-19 cases from this sequential pathway rises sharply from under 66% to over 74%. This finding supports the GSA result that ψ_c is a substantial amplifier of COVID-19 incidence.

In contrast, the influenza sequential pathway (bottom-right panel) shows very low sensitivity to ψ_f, remaining under 3% across the entire parameter space. The peak co-infection prevalence and cumulative co-infection (top panels) are most sensitive when both ψ_c and ψ_f are high (greater than 1.0).

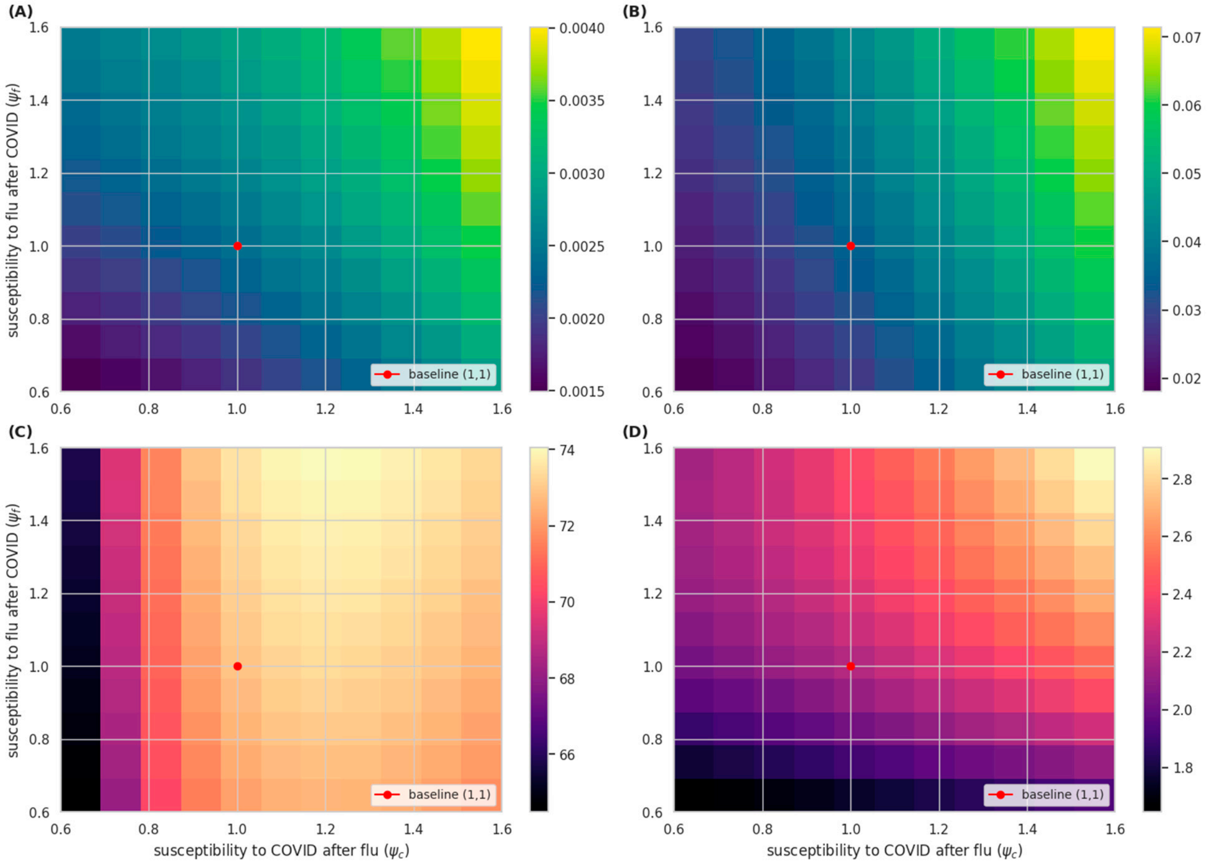

Figure S9. Sensitivity of co-epidemic outcomes to cross-susceptibility. The heatmaps show how four different outcomes change as ψ_c (susceptibility to COVID-19 after influenza, x-axis) and ψ_f (susceptibility to influenza after COVID-19, y-axis) are varied from 0.6 to 1.6. The red dot marks the baseline (ψ_c =1.0, ψ_f =1.0). **(A)** Peak co-infection prevalence (fraction). **(B)** Cumulative co-infection incidence over 180 days (fraction). **(C)** Share of total COVID-19 incidence from the sequential pathway (%). **(D)** Share of total influenza incidence from the sequential pathway (%).

References

[1] Mutua JM, Wang F-B, Vaidya NK. Modeling malaria and typhoid fever co-infection dynamics. Math Biosci 2015;264:128–44. https://doi.org/10.1016/j.mbs.2015.03.014.

[2] Shrestha S, King AA, Rohani P. Statistical Inference for Multi-Pathogen Systems. PLoS Comput Biol 2011;7:e1002135. https://doi.org/10.1371/journal.pcbi.1002135.

[3] Wong A, Barrero Guevara LA, Goult E, Briga M, Kramer SC, Kovacevic A, et al. The interactions of SARS-CoV-2 with cocirculating pathogens: Epidemiological implications and current knowledge gaps. PLoS Pathog 2023;19:e1011167. https://doi.org/10.1371/journal.ppat.1011167.

[4] Kramer SC, Pirikahu S, Casalegno J-S, Domenech de Cellès M. Characterizing the interactions between influenza and respiratory syncytial viruses and their implications for epidemic control. Nat Commun 2024;15:10066. https://doi.org/10.1038/s41467-024-53872-4.

[5] Dhakal M, Singh BK, Azad RK. Mechanistic Models of Virus–Bacteria Co-Infections in Humans: A Systematic Review of Methods and Assumptions. Pathogens 2025;14. https://doi.org/10.3390/pathogens14080830.

[6] Asamoah JKK, Okyere E, Abidemi A, Moore SE, Sun G-Q, Jin Z, et al. Optimal control and comprehensive cost-effectiveness analysis for COVID-19. Results Phys 2022;33:105177. https://doi.org/https://doi.org/10.1016/j.rinp.2022.105177.
[7] Alqarni MS, Alghamdi M, Muhammad T, Alshomrani AS, Khan MA. Mathematical modeling for novel coronavirus (COVID-19) and control. Numer Methods Partial Differ Equ 2022;38:760–76.
[8] Cori A, Valleron AJ, Carrat F, Scalia Tomba G, Thomas G, Boëlle PY. Estimating influenza latency and infectious period durations using viral excretion data. Epidemics 2012;4:132–8. https://doi.org/10.1016/j.epidem.2012.06.001.
[9] Avusuglo WS, Mosleh R, Ramaj T, Li A, Sharbayta SS, Fall AA, et al. Workplace absenteeism due to COVID-19 and influenza across Canada: A mathematical model. J Theor Biol 2023;572:111559. https://doi.org/10.1016/j.jtbi.2023.111559.
[10] Ojo MM, Benson TO, Peter OJ, Goufo EFD. Nonlinear optimal control strategies for a mathematical model of COVID-19 and influenza co-infection. Physica A: Statistical Mechanics and Its Applications 2022;607:128173. https://doi.org/10.1016/j.physa.2022.128173.
[11] Ngonghala CN, Gumel AB. 12 - Mathematical assessment of the role of vaccination against COVID-19 in the United States. In: Hernandez-Vargas EA, Velasco-Hernández JX, editors. Mathematical Modelling, Simulations, and AI for Emergent Pandemic Diseases, Academic Press; 2023, p. 221–49. https://doi.org/https://doi.org/10.1016/B978-0-323-95064-0.00013-0.
[12] Musa R, Peter OJ, Oguntolu FA. A non-linear differential equation model of COVID-19 and seasonal influenza co-infection dynamics under vaccination strategy and immunity waning. Healthcare Analytics 2023;4:100240. https://doi.org/10.1016/j.health.2023.100240.
[13] Saciuk Y, Kertes J, Mandel M, Hemo B, Shamir Stein N, Ekka Zohar A. Pfizer-BioNTech vaccine effectiveness against Sars-Cov-2 infection: Findings from a large observational study in Israel. Prev Med (Baltim) 2022;155:106947. https://doi.org/https://doi.org/10.1016/j.ypmed.2021.106947.
[14] Abidemi A, Fatoyinbo HO. Mathematical Analysis of Optimal Cost-Effective Control of COVID-19: A Case Study. 2021 International Conference on Decision Aid Sciences and Application (DASA), 2021, p. 95–102. https://doi.org/10.1109/DASA53625.2021.9682382.
